# Physical Mechanism of Spectra in Carbon Nanobelts under Quantum Size Effect

**DOI:** 10.3390/nano13010159

**Published:** 2022-12-29

**Authors:** Ning Li, Lei Zhang, Chen Lu, Ying Sun, Jingang Wang

**Affiliations:** 1 College of Science, Liaoning Petrochemical University, Fushun 113001, China; 2 Institute of Clean Energy Chemistry, Key Laboratory for Green Synthesis and Preparative Chemistry of Advanced Materials of Liaoning Province, College of Chemistry, Liaoning University, Shenyang 110036, China

**Keywords:** volume parameters, OPA, TPA, IR, Raman, nonlinear optical properties

## Abstract

Since the successful synthesis of [6,6]carbon nanobelt (CNB), [8,8]CNB and [12,12]CNB have been synthesized successively. CNBs with different sizes ([2N,2N]CNB; N = 2, 3, 4, 5, 6, 7, and 8) have quantum size effects and exhibit completely different optical properties. In this work, the linear and nonlinear optical properties and spectral changes of [2N,2N]CNB are studied based on density functional theory (DFT). The molecular volume, pore volume, and stability of [2N,2N]CNB are investigated. The electron transition mechanism of the one-photon absorption (OPA) and two-photon absorption (TPA) spectra of [2N,2N]CNB is explained, and the extrapolation formula between the wavelength of the absorption peak and the absorption coefficient (ε) and size is given. The infrared (IR) and Raman spectra of [2N,2N]CNB are calculated, and the vibrational modes of characteristic peaks are provided. Finally, the nonlinear optical properties of [2N,2N]CNB are studied, which reflect the anisotropy of molecular polarization. The extrapolation formulas for the polarizability (α) and second hyperpolarizability (γ) of [2N,2N]CNB under different external fields are given. The extrapolation formulas given in this work will help to predict the linear and nonlinear optical properties of arbitrary [2N,2N]CNB beyond computational power, laying the foundation for the practical application of [2N,2N]CNB’s theoretical basis.

## 1. Introduction

Carbon-based nanomaterials have attracted much attention due to their novel physical properties such as quantum size effects and surface effects [1,2,3,4,5]. With the development of nanomaterial-preparation technology and the improvement of observation methods, more and more nanoscale carbon-based materials (CNB, trigonenes, infinitene, cyclo[18]carbon, Möbius CNB, etc.) have been prepared [6,7,8,9]. Under the quantum size effect, they exhibit novel physical properties such as topological electronic features and deep ultraviolet emission regions. They have broad potential applications in quantum computing and topological materials for the future.

CNB composed of fully fused conjugated benzene rings have been a hot and difficult point in organic chemistry during the past 60 years [10]. By an iterative Wittig reaction followed by a nickel-mediated aryl–aryl coupling reaction, scientists successfully prepared circular-shaped CNBs that could be used as seeds for the preparation of carbon nanotubes [6]. Subsequently, CNBs of different scales were prepared [11]. These CNB materials exhibited strong quantum size effects and different spectroscopic properties, such as regular absorption, infrared, and Raman spectra. The highly delocalized electronic structure of sp2-hybridized graphene nanomaterials suggests their utility as optoelectronic and nonlinear optical materials [12,13,14,15]. Organic conjugated graphene nanomaterials have good applications in optoelectronic fields such as photoelectric sensors, photodetectors, and photovoltaic devices due to their strong charge-transfer ability [15]. Nonlinear optics is a broad field that describes the phenomena of elastic and inelastic light scattering when intense laser light interacts with materials. Nonlinear optics have great applications in optical imaging and sensing, optical switching and signal processing, biophotonics, and other fields [16,17,18,19].

**Figure 1:**
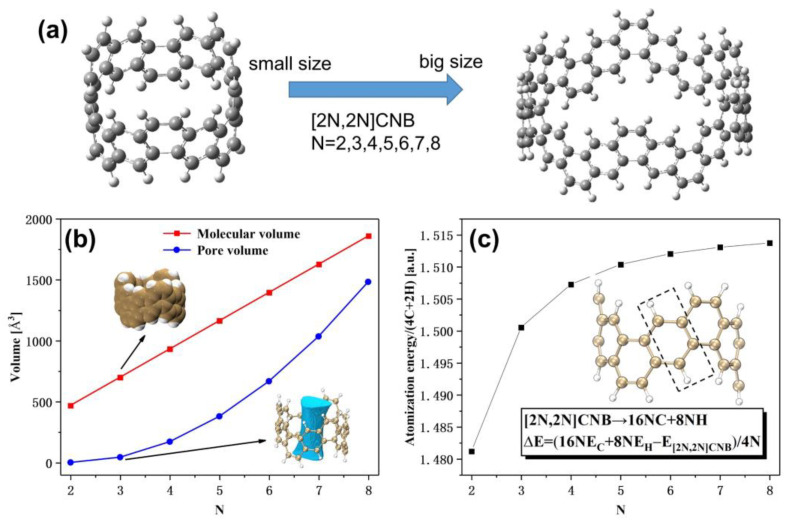
(**a**) Schematic diagram of the geometric structure of [2N,2N]CNB, with gray (white) spheres representing carbon (hydrogen) atoms. (**b**) Molecular volume and pore volume of [2N,2N]CNB. (**c**) The atomization energy per (4C + 2H) of [2N,2N]CNB. E_C_ and E_H_ are the electron energies of carbon and hydrogen atoms, respectively.

Here, we theoretically studied the OPA, TPA, IR, Raman, and (hyper)polarizability of CNBs with different sizes ([2N,2N]CNB; N = 2, 3, 4, 5, 6, 7, and 8; see Figure 1a) based on quantum chemical calculations and wave function analysis and explained the physical mechanism of light absorption by visualizing the charge-transfer process. Our research provides a theoretical basis for the application of CNB in optoelectronics, linear optics, and nonlinear optics.

## 2. Materials and Methods

The quantum chemical calculations for this work were completed by Gaussian 16 software [20]. We optimized the geometry of [2N,2N]CNB by density functional theory (DFT) [21], B3LYP functional [22], and def2-SVP basis set [23] combined with DFT-D3 dispersion correction [24]. Electron excitation spectra were calculated by the CAM-B3LYP functional [25] and the def2-SVP basis set. At the same time, the energy, dipole moment of each state, and transition dipole moment between each state used in the sum-over-states (SOS) [26] calculation all came from the electronic excitation calculation. The single-point energies used to calculate the atomization energy were calculated using the B3LYP functional and the def2-TZVP basis set. All wave-function analysis in this paper was completed by the multiwfn program [27], while 3D maps including molecular volume and pore volume were drawn by VMD [28].

## 3. Results

### 3.1. Study on Volume Parameters and Stability of [2N,2N]CNB

In this section, mainly the geometrical parameters including the molecular volume (Figure 1b) and pore volume (Figure 1c) of [2N,2N]CNB are investigated. This is very meaningful for understanding the interaction of [2N,2N]CNB with other substances. The definition of molecular volume is not unique, and the molecular volume calculated in this work is the van der Waals volume. The pore volume is defined as the superposition of the electron density of each atom in the free state, forming a promolecular density. This is equivalent to the state where atoms appear in the corresponding positions in the molecule, but the electron density has not yet been relaxed due to the distribution of bonds. The region within a certain isosurface of the excimer density is considered as the intramolecular region, and the region outside the isosurface can be regarded as the molecular hole. It can be seen that the molecular volume increases linearly. The reason is simple: the number of atoms in the system also grows linearly. However, the volume of the pores shows a nonlinear growth trend, while the curve shows a quadratic function.

The relative stability of [2N,2N]CNB is closely related to the possibility of its practical synthesis and application. Atomization energy is the energy change corresponding to the decomposition of a polyatomic molecule in the ground state of the gaseous state into atoms. It also corresponds to the energy released by the formation of covalent bonds between atoms and molecules, which can reflect the stability of the molecule. However, due to differing molecular sizes, direct comparisons cannot be made. Therefore, we evaluate the stability of [2N,2N]CNB by the atomization energy per (4C + 2H). It is not difficult to see that the atomization energy per (4C + 2H) increases gradually with the increase in [2N,2N]CNB (Figure 1c). This reflects the stronger stability of CNB with a large size. In addition, when N=5, the stability begins to gradually converge.

### 3.2. One-Photon Absorption Spectra of [2N,2N]CNB

**Figure 2:**
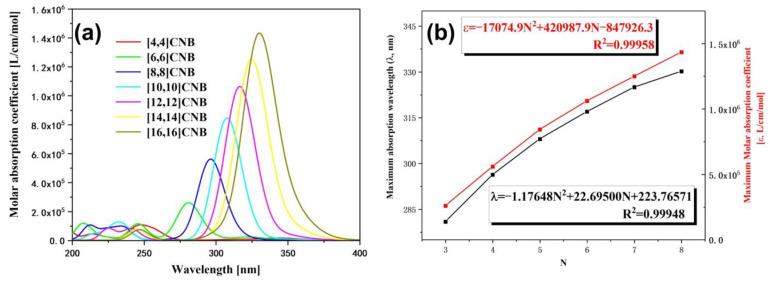
(**a**) OPA spectrum of [2N,2N]CNB. The absorption peak of the absorption spectrum is broadened by a Gaussian function, FWHM = 0.5 eV. (**b**) The relationship between the wavelength and ε of the maximum absorption peaks of [2N,2N]CNB and the size.

**Figure 3:**
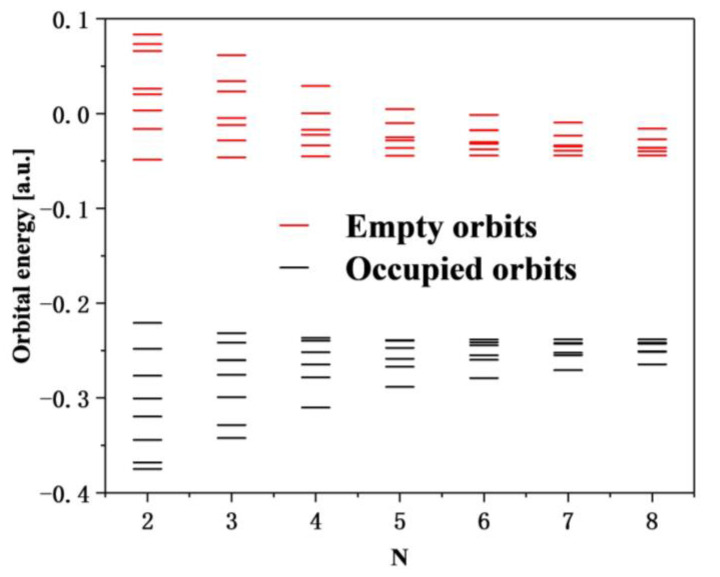
Energy levels of the 10 highest occupied orbitals and the 10 lowest empty orbitals in [2N,2N]CNB.

In this section, we calculate the UV–Vis absorption spectra of [2N,2N]CNB (Figure 2a). The strongest absorption peaks of [2N,2N]CNB all appear in the near-ultraviolet region. Moreover, with the increase in size, the strongest absorption peaks of [2N,2N]CNB gradually redshift. It is a common phenomenon that the absorption peaks of π-conjugated systems gradually redshift with an increase in size, which is caused by the increase in occupied orbital energy and the decrease in empty orbital energy [29]. The change in the energy levels of the 10 highest occupied orbitals and the 10 lowest empty orbitals of [2N,2N]CNB can be clearly seen in Figure 3. When N = 3, 4, 5, 6, 7, and 8, the wavelength of the strongest absorption peak of [2N,2N]CNB has strong regularity. We supply the orbital contribution of the excited states with the strongest oscillator of [2N,2N]CNB in the Supporting Information (Tables S1–S14). We complete a quadratic fit to the size and wavelength of [2N,2N]CNB, and the result is perfect ($R^{2}=0.99948$) (Figure 2b). This also shows that the wavelength of the strongest absorption peak of any size [2N,2N]CNB can be calculated according to this formula. To describe the electronic excitation characteristics of [2N,2N]CNBs with different sizes, we visualize the transition behavior of electrons by means of charge-density difference (CDD) maps [30,31,32,33]. The electron-hole density is defined as

(1) $$\rho^{hole}\left(r\right)={\sum_{i\to a}\left(\omega_{i}^{a}\right)}^{2}\varphi_{i}\varphi_{i}-{\sum_{i\leftarrow a}\left(\omega^{\prime }{}_{i}^{a}\right)}^{2}\varphi_{i}\varphi_{i}+\sum_{i\to a}\sum_{j\neq i\to a}\omega_{i}^{a}\omega_{j}^{a}\varphi_{i}\varphi_{j}-\sum_{i\leftarrow a}\sum_{j\neq i\leftarrow a}{\omega^{\prime }}_{i}^{a}{\omega^{\prime }}_{j}^{a}\varphi_{i}\varphi_{j}$$

(2) $$\rho^{ele}\left(r\right)={\sum_{i\to a}\left(\omega_{i}^{a}\right)}^{2}\varphi_{a}\varphi_{a}-{\sum_{i\leftarrow a}\left(\omega^{\prime }{}_{i}^{a}\right)}^{2}\varphi_{a}\varphi_{a}+\sum_{i\to a}\sum_{i\to b\neq a}\omega_{i}^{a}\omega_{i}^{b}\varphi_{a}\varphi_{b}-\sum_{i\leftarrow a}\sum_{i\leftarrow b\neq a}{\omega^{\prime }}_{i}^{a}{\omega^{\prime }}_{j}^{b}\varphi_{a}\varphi_{b}$$

where ω is the excitation configuration coefficient, and $\omega^{\prime }$ is the de-excitation configuration coefficient. r is the coordinate vector, φ is the orbital wave function, i or j is the occupied orbital label, and a or b is the empty orbital label. Thus, $\sum_{i\to a}$ represents every excitation configuration of the cycle, and $\sum_{i\leftarrow a}$ represents every de-excitation configuration of the cycle.

**Figure 4:**
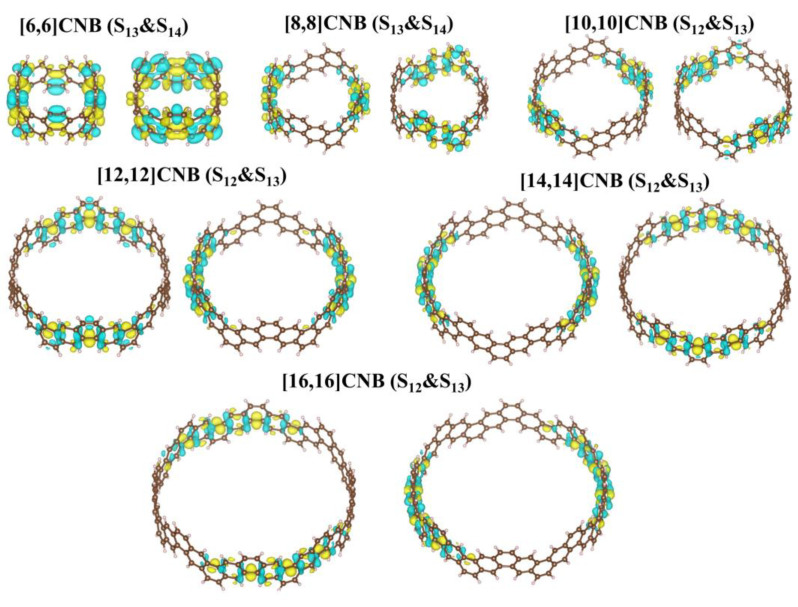
CDDs of the strongest absorption peaks of [2N,2N]CNB. Yellow and blue represent electrons and holes, respectively.

**Figure 5:**
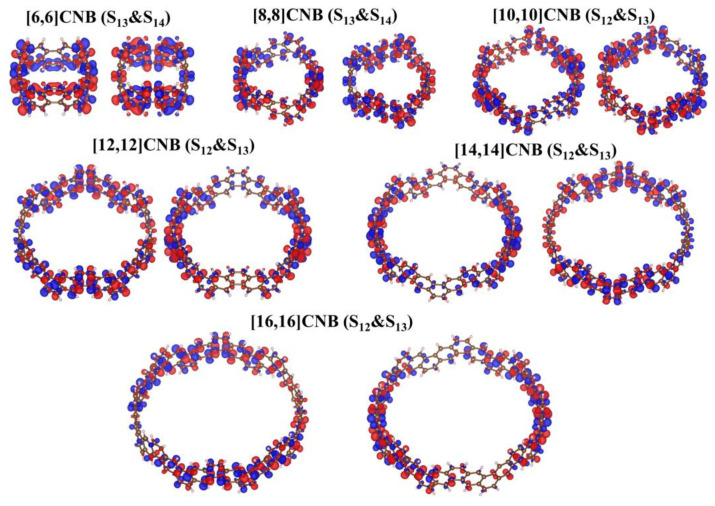
Transition dipole moment density of the strongest absorption peaks of [2N,2N]CNB. Red and blue represent the regions where the transition dipole moment is positive and negative, respectively.

The strongest absorption peaks of [2N,2N]CNB are both contributed to by a pair of degenerate excited states, and the pair of degenerate excited states are complementary in the transition region of the whole molecule. This is caused by the symmetry of [2N,2N]CNB. By observing the CDD diagrams, it can be found that the transitions corresponding to the maximum absorption peaks of [2N,2N]CNB with different sizes are $\pi \text{-}\pi^{*}$ transitions (Figure 4). The transition dipole moment density reflects the magnitude of the transition dipole moment, and the magnitude of the transition dipole moment is proportional to the oscillator strength of the excited state. It can be seen that the transition dipole moment density is mainly distributed on both sides of the [2N,2N]CNB (Figure 5) that corresponds to the CDD. In addition, with the increase in the size of [2N,2N]CNB, the transition dipole moment also increases gradually. This is also the reason why the ε of the strongest absorption peak increases gradually with the increase in the size of [2N,2N]CNB.

### 3.3. Two-Photon Absorption Spectra of [2N,2N]CNB

In this section, we calculated the two-photon absorption spectrum of [2N,2N]CNB and calculated the transition dipole moment between excited states through a script we wrote ourselves [34]. The two-photon molar absorptivity is defined as

(3) $$\begin{array}{l} \delta_{tp}=8\sum_{\begin{array}{l} j\neq g\\ j\neq f \end{array}}\frac{{\left|\langle f|\mu |j\rangle \right|}^{2}{\left|\langle j|\mu |g\rangle \right|}^{2}}{{\left(\omega_{j}-\omega_{f}/2\right)}^{2}+\Gamma_{f}^{2}}\left(1+2\cos^{2}\theta_{j}\right)\\ +8\frac{{\left|\Delta \mu_{fg}\right|}^{2}{\left|\langle f|\mu |g\rangle \right|}^{2}}{{\left(\omega_{f}/2\right)}^{2}+\Gamma_{f}^{2}}\left(1+2\cos^{2}\phi \right). \end{array}$$

where $|g\rangle$, $|j\rangle$, and $|f\rangle$ are the wave functions of the ground state, intermediate state, and final state during the TPA process, respectively.$\langle j|\mu |g\rangle$ and $\langle f|\mu |j\rangle$ are the transition dipole moments from the ground state to the intermediate state and the intermediate state to the final state, respectively. $\theta_{j}$ is the angle between the two transition dipole moments.$\Delta \mu_{fg}$ is the difference in the permanent dipole moments between the ground state and the final state. ϕ is the angle between $\Delta \mu_{fg}$ and $\langle f|\mu |g\rangle$.$\omega_{j}$ and $\omega_{f}$ are the energies of the intermediate state and the final state, respectively. $\Gamma_{f}$ is the lifetime of the ground state.

**Figure 6:**
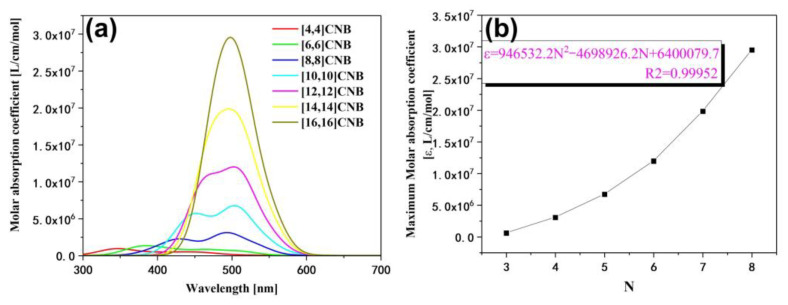
(**a**) TPA spectrum of [2N,2N]CNB. The absorption peak of the absorption spectrum is broadened by a Gaussian function, FWHM = 0.5 eV. (**b**) The relationship between the ε of the maximum absorption peaks of [2N,2N]CNB and the size.

**Figure 7:**
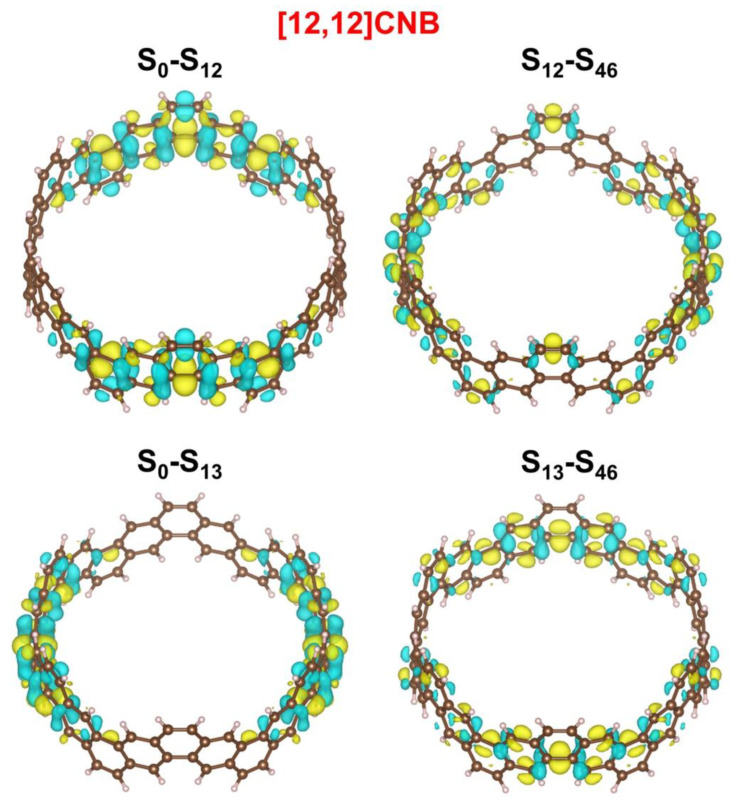
CDDs of the strongest TPA peaks of [2N,2N]CNB. Yellow and blue represent electrons and holes, respectively.

The strongest absorption peaks of the TPA spectra of [2N,2N]CNB are located at 500 nm, and the ε increases continuously with the increase in size (Figure 6a). The relationship between the size of [2N,2N]CNB and the maximum ε is also fitted (Figure 6b), and the curve is a quadratic function curve with a gradually increasing slope, which indicates that the ε gradually increased with the increase in size. Unlike the OPA spectrum, the TPA spectrum does not show a redshift of the absorption peak. By visualizing the charge transfer of the [2N,2N]CNB during the TPA process, it is found that the intermediate state in the transition process is the pair of degenerate states with the strongest oscillator intensity in the OPA spectrum. This is because this pair of degenerate states has the strongest transition dipole moment. Taking [2N,2N]CNB as an example, the strongest TPA peak of [2N,2N]CNB is contributed to by S_46_, S_12_, and S_13_, which are the intermediate states of S_46_. (Figure 7). The first-step transition of the two transition channels is the same as that of the OPA transition. The second-step transition is also a local excitation at both ends of the molecule, and the excitation region and the first-step transition are in a complementary state. The two-photon electronic transition processes of CNBs with different sizes are shown in Figures S1–S6. The second-step transitions of [6,6]CNB and [8,8]CNB are localized excitations located throughout the molecule. The two-photon transition process of [10,10]CNB, [14,14]CNB, and [16,16]CNB is the same as that of [12,12]CNB.

### 3.4. IR and Raman Spectra of [2N,2N]CNB

**Figure 8:**
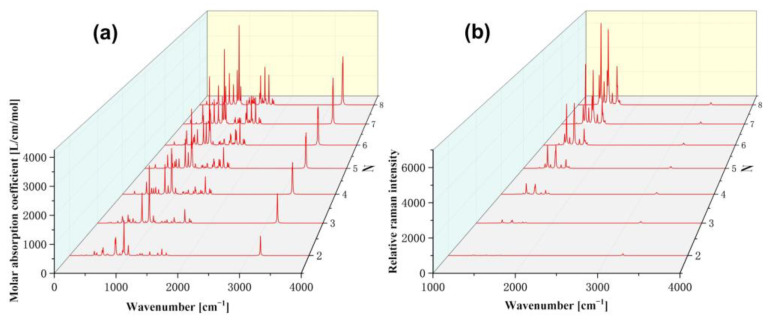
IR spectra (**a**) and Raman spectra (**b**) of [2N, 2N]CNB. The peaks of the infrared and Raman spectra are broadened by the Lorentz function, FWHM = 8.0 cm^−1^.

IR and Raman spectroscopy are common detection methods based on molecular vibrational modes, which can identify molecular types by the wavenumbers of characteristic peaks. The ε of the IR spectrum of [2N,2N]CNB gradually increases with the increase in size (Figure 8a,b). The IR spectrum of [2N,2N]CNB has more characteristic peaks in the range of 500–2000 cm^−1^, and the characteristic peak at 900 cm^−1^ is stronger. There is only one characteristic peak in the range of 2000–4000 cm^−1^, which is located at 3200 cm^−1^. The Raman spectrum has more characteristic peaks in the range of 1000–2000 cm^−1^, and the Raman intensity gradually increases with the increase in size. There is a characteristic peak with very small Raman intensity at 3200 cm^−1^, which does not change with the size of CNB.

**Figure 9:**
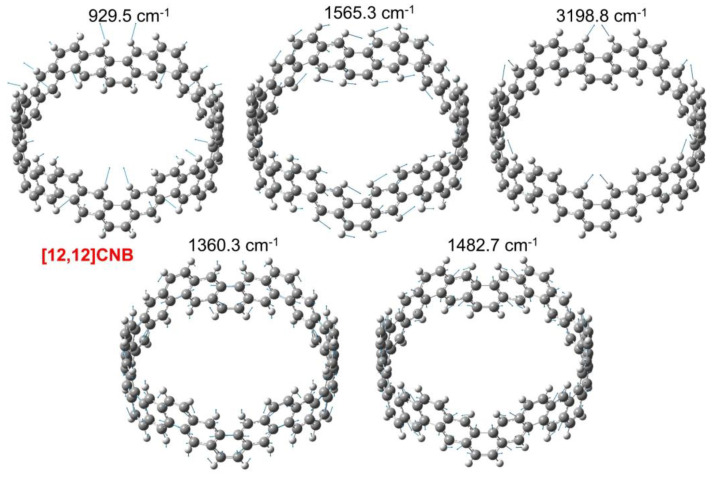
Vibration modes of [12,12]CNB at 929.5, 1360.3, 1482.7, 1565.3, and 3198.8 cm^−1^.

The vibrational modes of the larger characteristic peaks in the IR and Raman spectra of [12,12]CNB are shown in Figure 9. Among them, 925.5 cm^−1^, 1565.3 cm^−1^, and 3198.8 cm^−1^ have strong characteristic peaks in the IR spectrum, while 1360.3 cm^−1^ and 1482.7 cm^−1^ have strong characteristic peaks in the Raman spectrum. The vibrational mode of 925.5 cm^−1^ is that the hydrogen atom vibrates perpendicular to the circular molecular plane. The vibrational mode of 3198.8 cm^−1^ is the vibration of the hydrogen atom in the molecular circular plane. The vibration modes of 1565.3 cm^−1^, 1360.3 cm^−1^, and 1482.7 cm^−1^ are that carbon atoms and hydrogen atoms vibrate together in the molecular circular plane. The vibration modes of CNBs with different sizes are shown in Figures S7–S13, and the vibrational modes of the same wavenumber in different CNBs show similar displacement vectors.

### 3.5. Nonlinear Optical Properties of [2N,2N]CNB

In this section, we calculate the static/frequency-containing polarizability (α) and second hyperpolarizability (γ) of [2N,2N]CNB using the sum of states (SOS). Since [2N,2N]CNB is a centrosymmetric system, the first hyperpolarizability (β) is completely negligible. The SOS method is a common method for calculating (hyper)polarizability. The formulas for calculating α and γ are as follows:

(4) $$\alpha_{AB}(-\omega ;\omega )=\sum_{i\neq 0}\left[\frac{\mu_{0i}^{A}\mu_{i0}^{B}}{\Delta_{i}-\omega }+\frac{\mu_{0i}^{B}\mu_{i0}^{A}}{\Delta_{i}+\omega }\right]=\hat{P}\left[A\left(-\omega \right),B\left(\omega \right)\right]\sum_{i\neq 0}\frac{\mu_{0i}^{A}\mu_{i0}^{B}}{\Delta_{i}-\omega }.$$

(5) $$\gamma_{ABCD}\left(-\omega_{\sigma };\omega_{1},\omega_{2},\omega_{3}\right)=\hat{P}\left[A\left(-\omega_{\sigma }\right),B\left(\omega_{1}\right),C\left(\omega_{2}\right),D\left(\omega_{3}\right)\right]\left(\gamma^{I}-\gamma^{\mathrm{II}}\right).$$

(6) $$\gamma^{I}=\sum_{i\neq 0}\sum_{j\neq 0}\sum_{k\neq 0}\frac{\mu_{0i}^{A}\bar{\mu_{ij}^{B}\mu_{jk}^{C}}\mu_{k0}^{D}}{\left(\Delta_{i}-\omega_{\sigma }\right)\left(\Delta_{j}-\omega_{2}-\omega_{3}\right)\left(\Delta_{k}-\omega_{3}\right)}.$$

(7) $$\gamma^{\mathrm{II}}=\sum_{i\neq 0}\sum_{j\neq 0}\frac{\mu_{0i}^{A}\mu_{i0}^{B}\mu_{0j}^{C}\mu_{j0}^{D}}{\left(\Delta_{i}-\omega_{\sigma }\right)\left(\Delta_{i}-\omega_{1}\right)\left(\Delta_{j}-\omega_{3}\right)}.$$

(8) $$\mu_{ij}^{A}=\langle i|{\hat{\mu }}^{A}|j\rangle .$$

(9) $$\bar{\mu_{ij}^{A}}=\mu_{ij}^{A}-\mu_{00}^{A}\delta_{ij}.$$

(10) $$\omega_{\sigma }=\sum_{i}\omega_{i}.$$

where ω is the external field energy, and, when it is 0, it corresponds to the static (hyper)polarizability. Addition means summing over all the excited states. Δ is the excitation energy of the excited state relative to the ground state. P means to replace the items in square brackets. $u_{ij}^{A}$ represents the A-direction component of the transition dipole moment of the two states i and j. When i=j, the A-direction component corresponds to the dipole moment of the i-th state, so $u_{00}$ is the dipole moment of the ground state. $\delta_{ij}$ is the Kronecker symbol, which is 1 when i=j and 0 otherwise.

**Figure 10:**
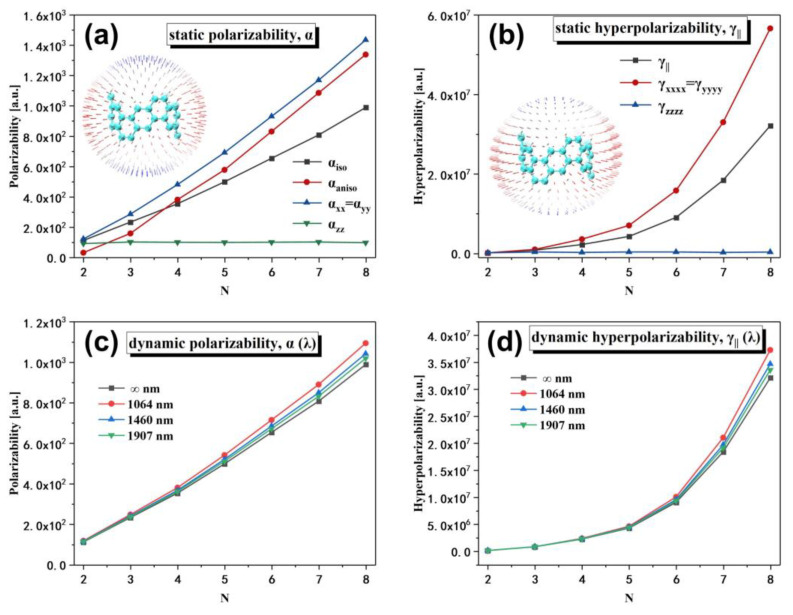
Static (**a**) and dynamic (**c**) polarizability of [2N,2N]CNB; static (**b**) and dynamic (**d**) second hyperpolarizability of [2N,2N]CNB.

The polarizability of [2N,2N]CNB shows obvious anisotropy ($\alpha_{\mathrm{aniso}}$), which is due to the increase in the size of [2N,2N]CNB along the xy direction (Figure 10a). This results in the delocalized π electrons on the [2N,2N]CNB having a wider delocalized space in the xy plane under the action of the external field. As the size increases, the isotropic average polarizability ($\alpha_{\mathrm{iso}}$) of the CNBs gradually becomes larger. Studies have shown that the polarizability of the system is positively correlated with the volume [35], which is also consistent with our conclusion. $\alpha_{x}$ and $\alpha_{y}$ are equal and grow gradually with size. However, $\alpha_{z}$ does not change significantly with the increase in the size of [2N,2N]CNB. This is because the size of [2N,2N]CNB does not change in the z direction. Like the polarizability, the γ of [2N,2N]CNB also exhibits strong anisotropy (Figure 10b). In addition, with the increase in size, the enhancement of the γ is obvious, showing a nonlinear trend. From the unit sphere of the static (second hyper)polarizability tensor, it can be seen that the static (second hyper)polarizability at the xy plane reaches a maximum value, and, as the angle to the xy plane increases, the static (second hyper)polarizability gradually decreases. Next, we calculated the dynamic α and γ of [2N,2N]CNB at external fields of 1064, 1460, and 1907 nm. It can be seen that the frequency-containing α and γ of [2N,2N]CNB gradually increase with the enhancement of the external field (Figure 10c,d). It is well understood that the stronger the external field is, the more polarized the electrons will be. To clarify the relationship between the size of [2N,2N]CNB and the (second super)polarizability, we separately fit the function of the α and γ as a function (Table 1 and Table 2) of size under different external fields (1064, 1460, 1907, and ∞ nm). The fitting results are very satisfactory, with $R^{2}\;>\;0.999$ for all functions. The excellent fitting results can be used to predict the static/dynamic α and γ of [2N,2N]CNBs of arbitrary size.

## 4. Conclusions

In this work, the geometric structure, stability, photophysical properties, and nonlinear optical properties of [2N,2N]CNB (N = 2, 3, 4, 5, 6, 7, and 8) are theoretically investigated. As the size of [2N,2N]CNB increases, the system becomes increasingly stable. In addition, when N=5, the stability of [2N,2N]CNB gradually converges. The maximum absorption peak of the OPA spectrum of [2N,2N]CNB gradually redshifted with the increase in size, and the absorption intensity gradually increased. The maximum absorption peak is contributed to by a pair of degenerate excited states, and the electron transition of this pair of degenerate excited states is localized excitation at both ends of the molecule. The peak position of the maximum absorption peak of the TPA spectrum does not change with the size, and the absorptivity increases gradually. The intensities of the characteristic peaks of the IR and Raman spectra gradually increase with the increase in size. The α and γ of [2N,2N]CNB increase significantly with increasing size and show a pronounced anisotropy. The value of the α and γ parallel to the xy plane reaches a maximum value, while the value of the α and γ perpendicular to the xy plane is minimal. In addition, the α and γ of [2N,2N]CNB gradually increase with the enhancement of the external field. These properties reflect a very strong regularity. We fit equations for the wavelength of the strongest absorption peak, α, and γ for different external fields as a function of the size of [2N,2N]CNB. The relevant properties of large-scale [2N,2N]CNB beyond computational power can be reliably obtained by these equations.

## Figures and Tables

**Table 1:** The extrapolation formula of the polarizability of [2N,2N]CNB under different external fields.

Field Frequency (λ, nm)	$\alpha_{\mathrm{iso}}=AN^{2}+BN+C$			$R^{2}$
	A	B	C	
∞	5.82	87.72	−86.80	0.99988
1064	7.25	90.08	−90.62	0.99990
1460	6.53	89.08	−89.44	0.99989
1907	6.22	88.54	−88.36	0.99988

**Table 2:** The extrapolation formula of the second hyperpolarizability of [2N,2N]CNB under different external fields.

Field Frequency (λ, nm)	$\gamma_{\left|\right|}=AN^{3}+BN^{2}+CN+D$				$R^{2}$
	A	B	C	D	
∞	211,176.32	−1,854,522.24	6,157,267.00	−6,525,686.13	0.99973
1064	257,845.76	−2,303,081.44	7,590,780.84	−7,991,560.47	0.99971
1460	234,094.08	−2,073,731.36	6,857,161.72	−7,241,207.30	0.99972
1907	224,148.48	−1,978,346.88	6,552,515.92	−6,929,772.45	0.99973

## Data Availability

Not applicable.

## Supplementary Materials

The following supporting information can be downloaded at: https://www.mdpi.com/article/10.3390/nano13010159/s1. Figure S1: CDD of S_0_→S_13_→S_24_ and S_0_→S_14_→S_24_ in the TPA of [6,6]CNB. Figure S2. CDD of S0→S13→S39 and S0→S14→S39 in the TPA of [8,8]CNB. Figure S3. CDD of S0→S12→S42 and S0→S13→S42 in the TPA of [10,10]CNB. Figure S4. CDD of S0→S12→S46 and S0→S13→S46 in the TPA of [12,12]CNB. Figure S5. CDD of S0→S12→S53 and S0→S13→S53 in the TPA of [14,14]CNB. Figure S6. CDD of S0→S14→S56 and S0→S15→S56 in the TPA of [16,16]CNB. Figure S7. Vibrational modes of [4,4]CNB at 908.0, 1312.4, 1477.2, 1540.7 and 3190.1 cm^−1^. Figure S8. Vibrational modes of [6,6]CNB at 918.2, 1344.7, 1479.3, 1548.1 and 3193.1 cm^−1^. Figure S9. Vibrational modes of [8,8]CNB at 921.6, 1354.0, 1480.9, 1556.6 and 3196.1 cm^−1^. Figure S10. Vibrational modes of [10,10]CNB at 926.6, 1358.2, 1482.0, 1562.0 and 3197.9 cm^−1^. Figure S11. Vibrational modes of [12,12]CNB at 929.5, 1360.3, 1482.7, 1565.3 and 3198.8 cm^−1^. Figure S12. Vibrational modes of [14,14]CNB at 903.9, 1390.3, 1497.1, 1515.9 and 3286.6 cm^−1^. Figure S13. Vibrational modes of [16,16]CNB at 905.6, 1391.5, 1498.5, 1516.4 and 3287.0 cm^−1^; Table S1. Orbital contribution of S_18_ of [4,4]CNB. Table S2. Orbital contribution of S19 of [4,4]CNB. Table S3. Orbital contribution of S13 of [6,6]CNB. Table S4. Orbital contribution of S_14_ of [6,6]CNB. Table S5. Orbital contribution of S_13_ of [8,8]CNB. Table S6. Orbital contribution of S14 of [8,8]CNB. Table S7. Orbital contribution of S12 of [10,10]CNB. Table S8. Orbital contribution of S13 of [10,10]CNB. Table S9. Orbital contribution of S12 of [12,12]CNB. Table S10. Orbital contribution of S13 of [12,12]CNB. Table S11. Orbital contribution of S12 of [14,14]CNB. Table S12. Orbital contribution of S13 of [14,14]CNB. Table S13. Orbital contribution of S12 of [16,16]CNB. Table S14. Orbital contribution of S13 of [16,16]CNB.
