# Supplementary material for: Physical Mechanism of Spectra in Carbon Nanobelts under Quantum Size Effect
Source: Nanomaterials (Basel). 2022 Dec 29;13(1):159. doi: 10.3390/nano13010159 (PMC9823666; doi:10.3390/nano13010159)
Supplement: Supplementary file 1 [file nanomaterials-13-00159-s001.zip › nanomaterials-2106559-SI.pdf]

nanomaterials

Article

Physical Mechanism of Spectra in Carbon Nanobelts under Quantum Size Effect

Ning Li [1,†], Lei Zhang [1,*], Chen Lu [1,†], Ying Sun [2,*] and Jingang Wang [1,*]

[1] College of Science, Liaoning Petrochemical University, Fushun 113001, China
[2] Institute of Clean Energy Chemistry, Key Laboratory for Green Synthesis and Preparative Chemistry of Advanced Materials of Liaoning Province, College of Chemistry, Liaoning University, Shenyang 110036, China
* Correspondence: zhanglei@lnpu.edu.cn (L.Z.); yingsun@lnu.edu.cn (Y.S.); jingang_wang@lnpu.edu.cn (J.W.)
† These authors contributed equally to this work.

Supplementary Materials

Table S1. Orbital contribution of S_{18} of [4,4]CNB

[4,4]CNB&S_{18}				
occupied orbit		empty orbit	Configuration factor	%
HOMO-2	→	LUMO+1	0.60094	72.23%
HOMO-4	→	LUMO	-0.25055	12.56%
HOMO	→	LUMO+6	0.14734	4.34%
HOMO-3	→	LUMO+4	0.13203	3.49%
HOMO-1	→	LUMO+5	0.09645	1.86%
HOMO-3	→	LUMO	0.07123	1.01%
HOMO-6	→	LUMO+6	0.06094	0.74%
HOMO-2	→	LUMO+7	0.05115	0.52%
HOMO-5	→	LUMO+1	0.04239	0.36%
HOMO-4	→	LUMO+4	0.03961	0.31%

Table S2. Orbital contribution of S_{19} of [4,4]CNB.

[4,4]CNB&S_{19}				
occupied orbit		empty orbit	Configuration factor	%
HOMO-3	→	LUMO+1	0.60095	72.23%
HOMO-5	→	LUMO	-0.25053	12.55%
HOMO	→	LUMO+5	0.14729	4.34%
HOMO-2	→	LUMO+4	0.13206	3.49%
HOMO-1	→	LUMO+6	0.09649	1.86%
HOMO-2	→	LUMO	0.07121	1.01%
HOMO-6	→	LUMO+5	0.06095	0.74%
HOMO-3	→	LUMO+7	0.05115	0.52%
HOMO-4	→	LUMO+1	0.04236	0.36%
HOMO-5	→	LUMO+4	0.03959	0.31%

Table S3. Orbital contribution of S_{13} of [6,6]CNB

[6,6]CNB&S_{13}				
occupied orbit		empty orbit	Configuration factor	%
HOMO-1	→	LUMO+2	0.34023	23.15%
HOMO-2	→	LUMO+1	0.33771	22.81%
HOMO-5	→	LUMO	-0.31329	19.63%
HOMO	→	LUMO+5	-0.26725	14.28%
HOMO-7	→	LUMO	-0.22025	9.70%
HOMO-3	→	LUMO+4	-0.11019	2.43%
HOMO-2	→	LUMO+5	0.11012	2.43%
HOMO-2	→	LUMO+7	0.0698	0.97%
HOMO-3	→	LUMO+6	-0.06978	0.97%
HOMO-8	→	LUMO+5	0.06509	0.85%

Table S4. Orbital contribution of S_{14} of [6,6]CNB

[6,6]CNB&S_{14}				
occupied orbit		empty orbit	Configuration factor	%
HOMO-1	→	LUMO+3	0.34058	23.20%
HOMO-3	→	LUMO+1	-0.33801	22.85%
HOMO-4	→	LUMO	0.31276	19.56%
HOMO	→	LUMO+4	0.26723	14.28%
HOMO-6	→	LUMO	-0.22022	9.70%
HOMO-2	→	LUMO+4	0.10999	2.42%
HOMO-3	→	LUMO+5	0.10988	2.41%
HOMO-2	→	LUMO+6	0.06989	0.98%
HOMO-3	→	LUMO+7	0.06983	0.98%
HOMO-8	→	LUMO+4	-0.06502	0.85%

Table S5. Orbital contribution of S_{13} of [8,8]CNB

[8,8]CNB&S_{13}				
occupied orbit		empty orbit	Configuration factor	%
HOMO	→	LUMO+4	0.34952	24.43%
HOMO-1	→	LUMO+2	-0.3411	23.27%
HOMO-3	→	LUMO+1	0.32875	21.62%
HOMO-5	→	LUMO	-0.32033	20.52%
HOMO-4	→	LUMO+6	0.07057	1.00%
HOMO-5	→	LUMO+7	-0.07019	0.99%
HOMO	→	LUMO+8	-0.06939	0.96%
HOMO-3	→	LUMO+7	0.06892	0.95%
HOMO-2	→	LUMO+6	0.06891	0.95%
HOMO-7	→	LUMO+2	-0.06191	0.77%

Table S6. Orbital contribution of S_{14} of [8,8]CNB

		[8,8]CNB&S_{14}		
occupied orbit		empty orbit	Configuration factor	%
HOMO	→	LUMO+5	0.34952	24.43%
HOMO-	→	LUMO+3	-0.3411	23.27%
HOMO-2	→	LUMO+1	0.32875	21.62%
HOMO-4	→	LUMO	-0.32033	20.52%
HOMO-5	→	LUMO+6	0.07057	1.00%
HOMO-4	→	LUMO+7	0.07019	0.99%
HOMO	→	LUMO+9	-0.06939	0.96%
HOMO-2	→	LUMO+7	-0.06892	0.95%
HOMO-3	→	LUMO+6	0.06891	0.95%
HOMO-7	→	LUMO+3	0.06191	0.77%

Table S7. Orbital contribution of S_{12} of [10,10]CNB

		[10,10]CNB&S_{12}		
occupied orbit		empty orbit	Configuration factor	%
HOMO-4	→	LUMO	0.35061	24.59%
HOMO-1	→	LUMO+4	0.34874	24.32%
HOMO	→	LUMO+3	-0.28997	16.82%
HOMO-3	→	LUMO+1	-0.27376	14.99%
HOMO-2	→	LUMO+1	-0.10913	2.38%
HOMO-2	→	LUMO+6	-0.07959	1.27%
HOMO-3	→	LUMO+7	-0.07926	1.26%
HOMO-6	→	LUMO+2	-0.07148	1.02%
HOMO-7	→	LUMO+3	-0.07119	1.01%
HOMO-1	→	LUMO+5	0.07071	1.00%

Table S8. Orbital contribution of S_{13} of [10,10]CNB

		[10,10]CNB&S_{13}		
occupied orbit		empty orbit	Configuration factor	%
HOMO-5	→	LUMO	0.35103	24.64%
HOMO-1	→	LUMO+5	-0.3492	24.39%
HOMO	→	LUMO+2	0.28951	16.76%
HOMO-2	→	LUMO+1	0.27328	14.94%
HOMO-3	→	LUMO+1	-0.10916	2.38%
HOMO-3	→	LUMO+6	0.07913	1.25%
HOMO-2	→	LUMO+7	-0.07905	1.25%
HOMO-6	→	LUMO+3	0.07104	1.01%
HOMO-7	→	LUMO+2	-0.071	1.01%
HOMO-1	→	LUMO+4	0.07055	1.00%

Table S9. Orbital contribution of S_{12} of [12,12]CNB

		[12,12]CNB&S_{12}		
occupied orbit		empty orbit	Configuration factor	%
HOMO-5	→	LUMO	0.36741	27.00%
HOMO-1	→	LUMO+4	-0.36474	26.61%
HOMO	→	LUMO+2	-0.25591	13.10%
HOMO-3	→	LUMO+1	-0.25455	12.96%
HOMO-4	→	LUMO+8	-0.1093	2.39%
HOMO-5	→	LUMO+9	-0.10868	2.36%
HOMO-10	→	LUMO+5	0.10862	2.36%
HOMO-11	→	LUMO+4	-0.108	2.33%
HOMO-3	→	LUMO+6	-0.09407	1.77%
HOMO-2	→	LUMO+7	0.09352	1.75%

Table S10. Orbital contribution of S_{13} of [12,12]CNB

		[12,12]CNB&S_{13}		
occupied orbit		empty orbit	Configuration factor	%
HOMO-4	→	LUMO	0.36741	27.00%
HOMO-1	→	LUMO+5	-0.36474	26.61%
HOMO	→	LUMO+3	-0.25591	13.10%
HOMO-2	→	LUMO+1	0.25455	12.96%
HOMO-5	→	LUMO+8	-0.1093	2.39%
HOMO-4	→	LUMO+9	0.10868	2.36%
HOMO-10	→	LUMO+4	0.10862	2.36%
HOMO-11	→	LUMO+5	0.108	2.33%
HOMO-2	→	LUMO+6	-0.09407	1.77%
HOMO-3	→	LUMO+7	-0.09352	1.75%

Table S11. Orbital contribution of S_{12} of [14,14]CNB

		[14,14]CNB&S_{12}		
occupied orbit		empty orbit	Configuration factor	%
HOMO-1	→	LUMO+4	0.34196	23.39%
HOMO-4	→	LUMO	-0.3159	19.96%
HOMO	→	LUMO+3	0.21728	9.44%
HOMO-5	→	LUMO	-0.19573	7.66%
HOMO-3	→	LUMO+1	0.1928	7.43%
HOMO-1	→	LUMO+5	-0.13817	3.82%
HOMO-10	→	LUMO+5	-0.12602	3.18%
HOMO-11	→	LUMO+4	-0.12599	3.17%
HOMO-5	→	LUMO+8	0.10291	2.12%
HOMO-4	→	LUMO+9	-0.10289	2.12%

Table S12. Orbital contribution of S_{13} of [14,14]CNB

[14,14]CNB&S_{13}				
occupied orbit		empty orbit	Configuration factor	%
HOMO-1	→	LUMO+5	0.34197	23.39%
HOMO-5	→	LUMO	-0.31591	19.96%
HOMO	→	LUMO+2	-0.21727	9.44%
HOMO-4	→	LUMO	0.19573	7.66%
HOMO-2	→	LUMO+1	-0.19279	7.43%
HOMO-1	→	LUMO+4	0.13817	3.82%
HOMO-10	→	LUMO+4	-0.12603	3.18%
HOMO-11	→	LUMO+5	0.12599	3.17%
HOMO-4	→	LUMO+8	0.10293	2.12%
HOMO-5	→	LUMO+9	0.10289	2.12%

Table S13. Orbital contribution of S_{12} of [16,16]CNB

[16,16]CNB&S_{12}				
occupied orbit		empty orbit	Configuration factor	%
HOMO-8	→	LUMO	0.27239	14.84%
HOMO-6	→	LUMO+4	0.20413	8.33%
HOMO-7	→	LUMO+5	-0.20412	8.33%
HOMO-3	→	LUMO+10	-0.19954	7.96%
HOMO-2	→	LUMO+9	0.14906	4.44%
HOMO-1	→	LUMO+8	0.14906	4.44%
HOMO-5	→	LUMO+6	0.12373	3.06%
HOMO-4	→	LUMO+7	0.12371	3.06%
HOMO-13	→	LUMO+5	0.10307	2.12%
HOMO-12	→	LUMO+4	-0.10307	2.12%

Table S14. Orbital contribution of S_{13} of [16,16]CNB

[16,16]CNB&S_{13}				
occupied orbit		empty orbit	Configuration factor	%
HOMO-9	→	LUMO	0.27239	14.84%
HOMO-6	→	LUMO+5	0.20413	8.33%
HOMO-7	→	LUMO+4	0.20412	8.33%
HOMO-3	→	LUMO+11	-0.19954	7.96%
HOMO-1	→	LUMO+9	-0.14906	4.44%
HOMO-2	→	LUMO+8	0.14906	4.44%
HOMO-4	→	LUMO+6	-0.12373	3.06%
HOMO-5	→	LUMO+7	0.12371	3.06%
HOMO-13	→	LUMO+4	0.10307	2.12%
HOMO-12	→	LUMO+5	0.10307	2.12%

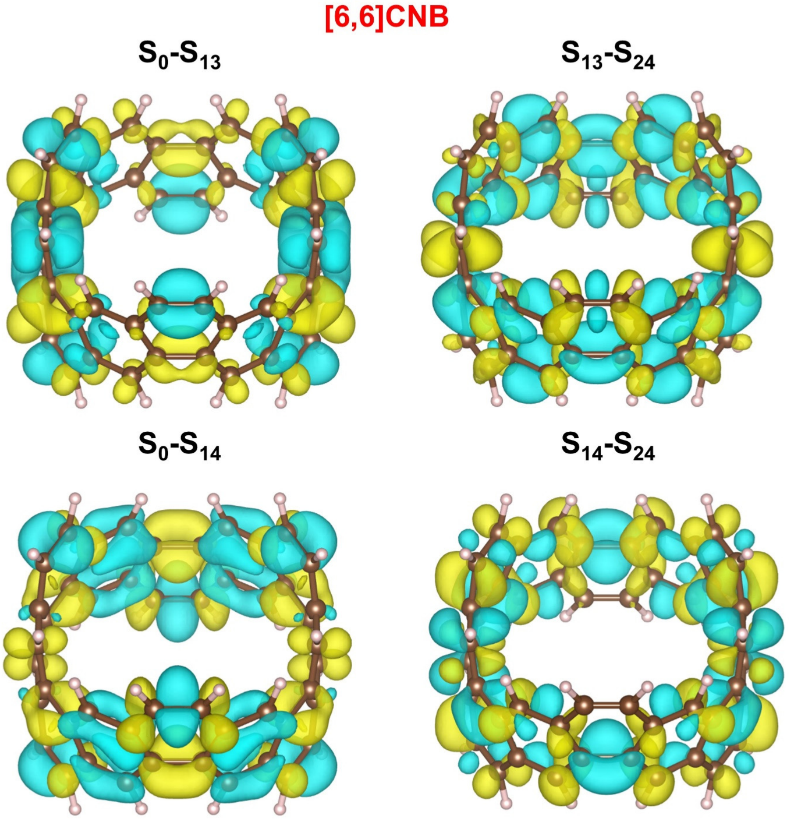

Figure S1. CDD of $S_0 \rightarrow S_{13} \rightarrow S_{24}$ and $S_0 \rightarrow S_{14} \rightarrow S_{24}$ in the TPA of [6,6]CNB.

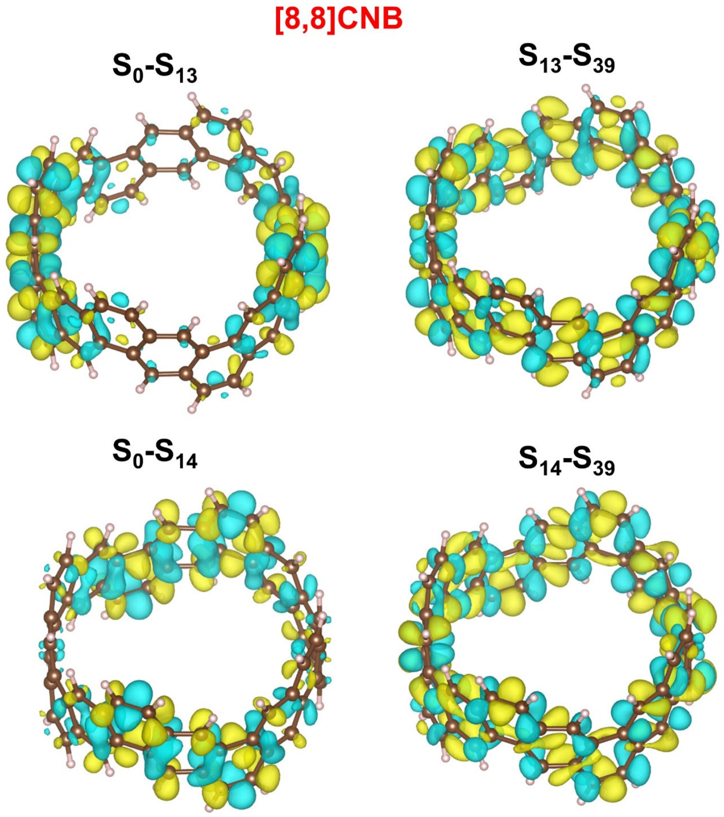

Figure S2. CDD of $S_0 \rightarrow S_{13} \rightarrow S_{39}$ and $S_0 \rightarrow S_{14} \rightarrow S_{39}$ in the TPA of [8,8]CNB.

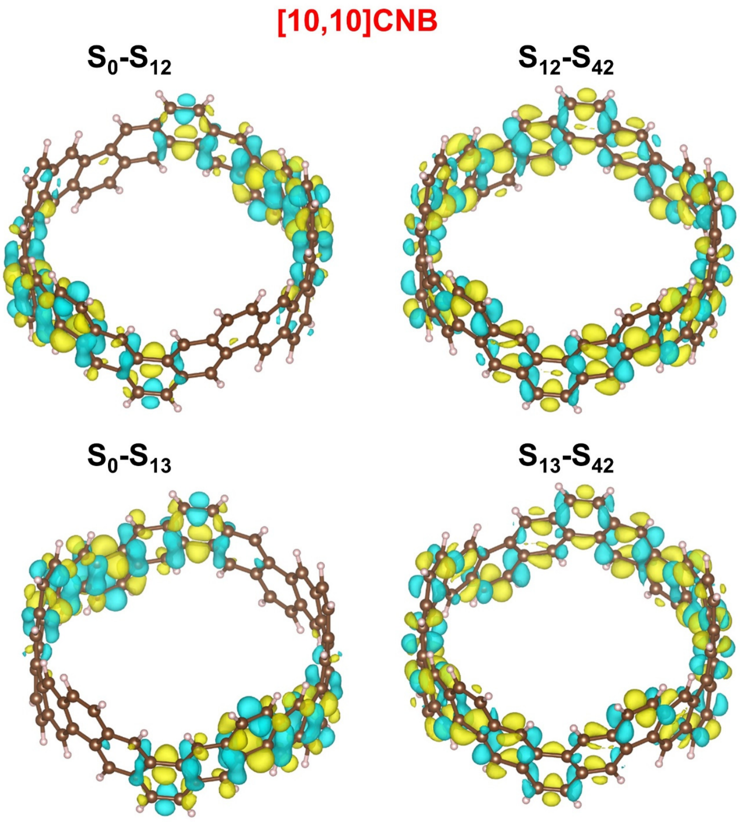

Figure S3. CDD of $S_0 \rightarrow S_{12} \rightarrow S_{42}$ and $S_0 \rightarrow S_{13} \rightarrow S_{42}$ in the TPA of [10,10]CNB.

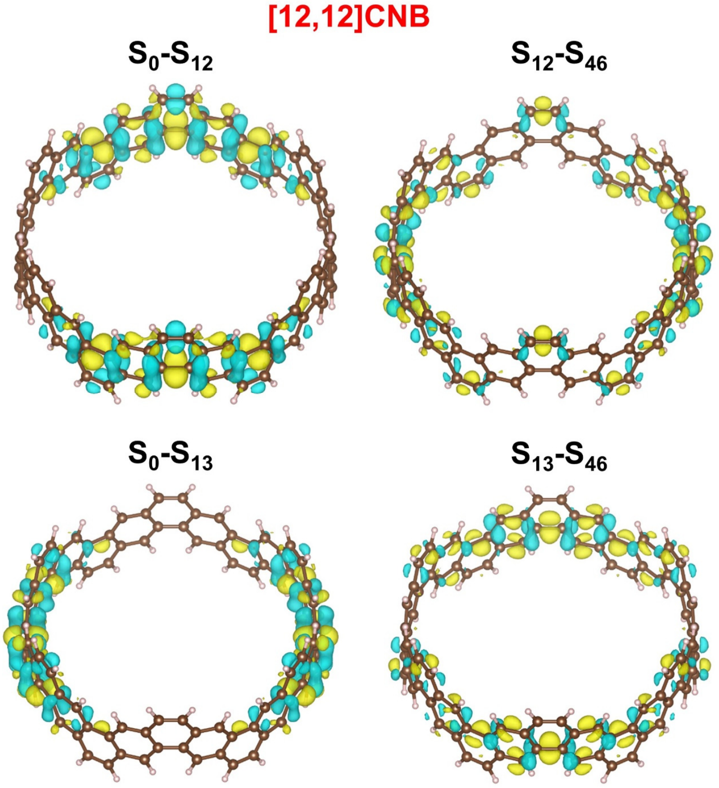

Figure S4. CDD of $S_0 \rightarrow S_{12} \rightarrow S_{46}$ and $S_0 \rightarrow S_{13} \rightarrow S_{46}$ in the TPA of [12,12]CNB.

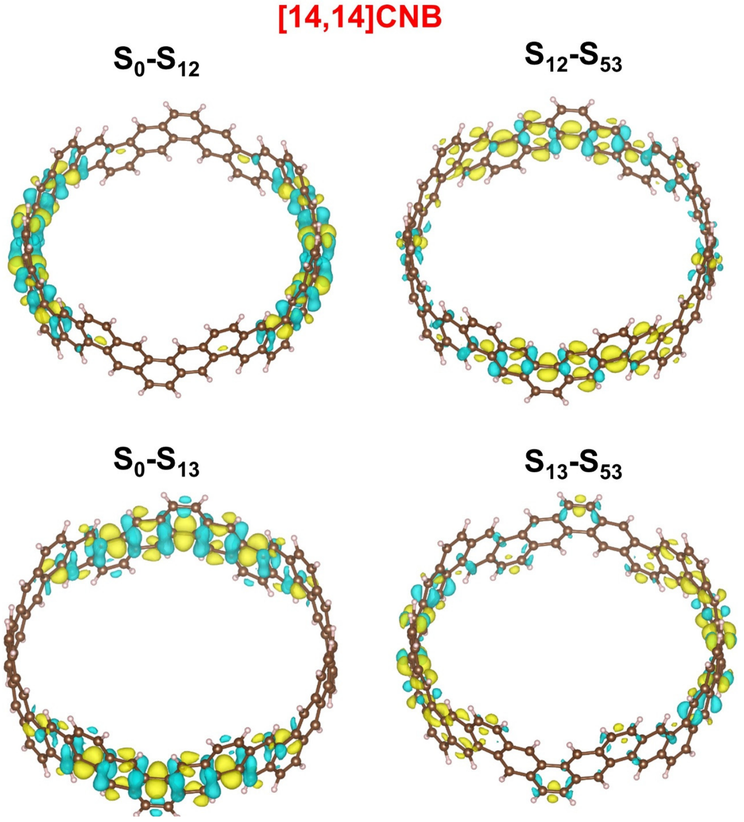

Figure S5. CDD of $S_0 \rightarrow S_{12} \rightarrow S_{53}$ and $S_0 \rightarrow S_{13} \rightarrow S_{53}$ in the TPA of [14,14]CNB.

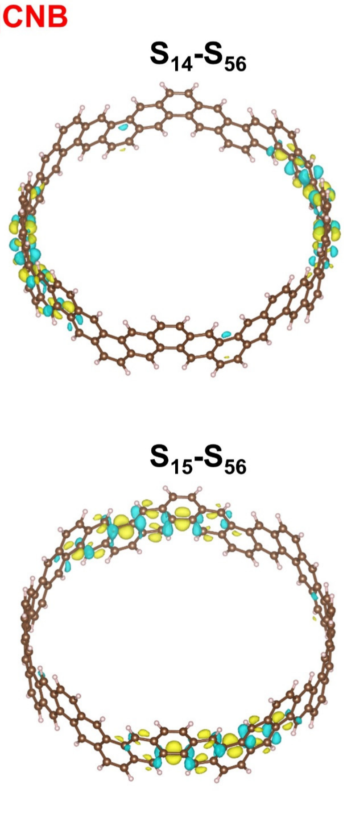

Figure S6. CDD of $S_0 \rightarrow S_{14} \rightarrow S_{56}$ and $S_0 \rightarrow S_{15} \rightarrow S_{56}$ in the TPA of [16,16]CNB.

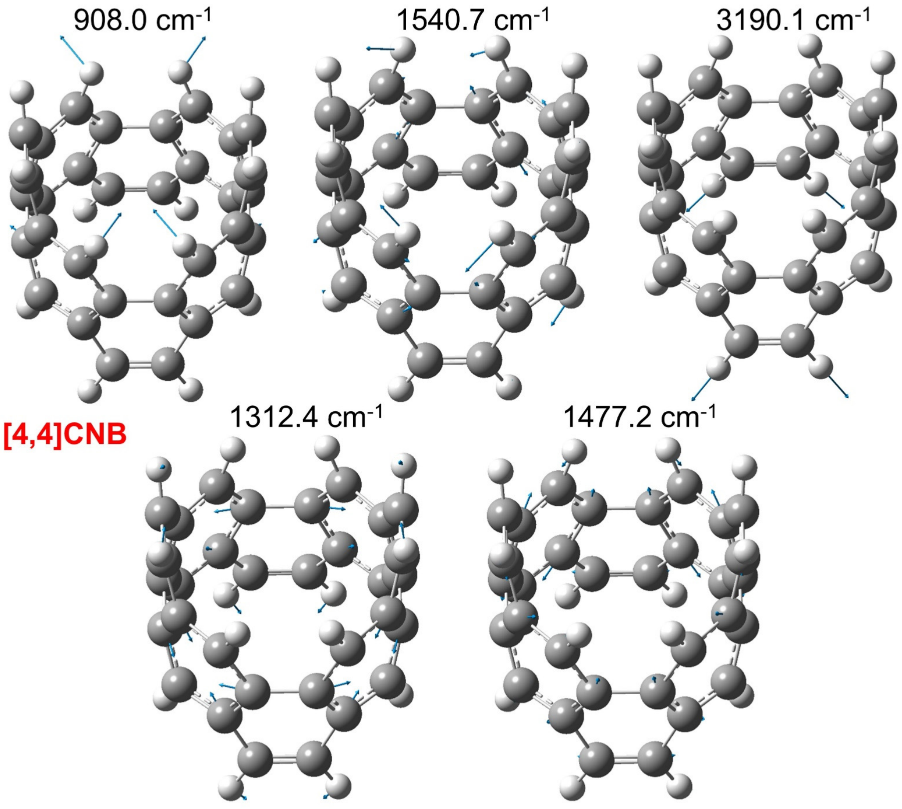

Figure S7. Vibrational modes of [4,4]CNB at 908.0, 1312.4, 1477.2, 1540.7 and 3190.1 cm^{-1}

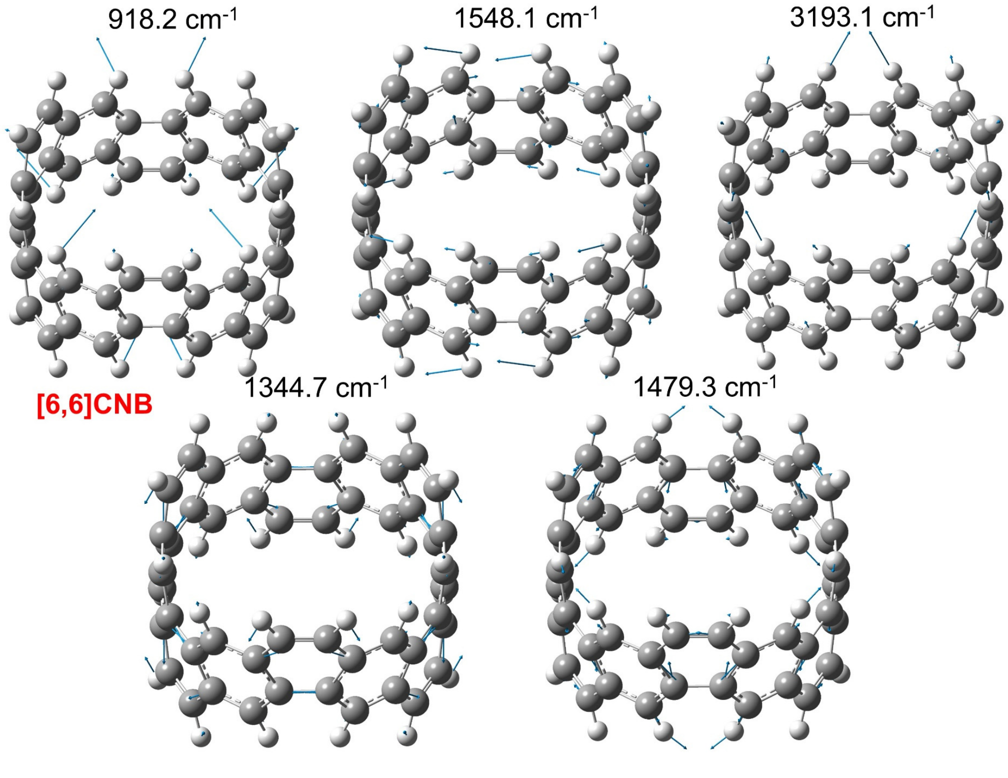

Figure S8. Vibrational modes of [6,6]CNB at 918.2, 1344.7, 1479.3, 1548.1 and 3193.1 cm^{-1}

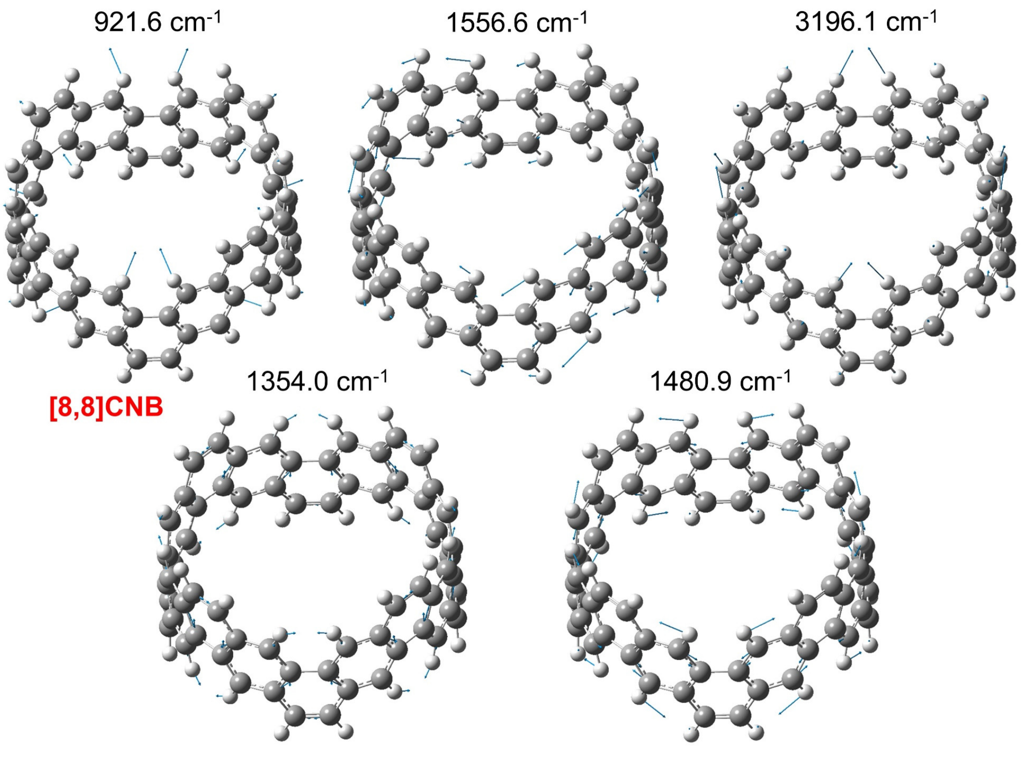

Figure S9. Vibrational modes of [8,8]CNB at 921.6, 1354.0, 1480.9, 1556.6 and 3196.1 cm^{-1}

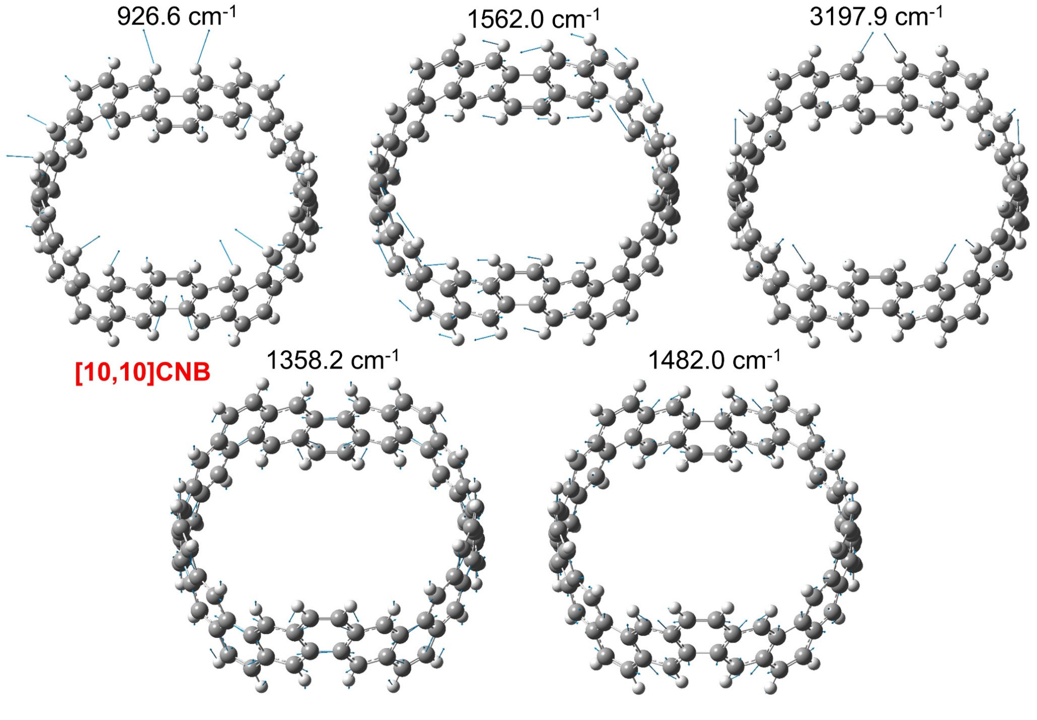

Figure S10. Vibrational modes of [10,10]CNB at 926.6, 1358.2, 1482.0, 1562.0 and 3197.9 cm^{-1}

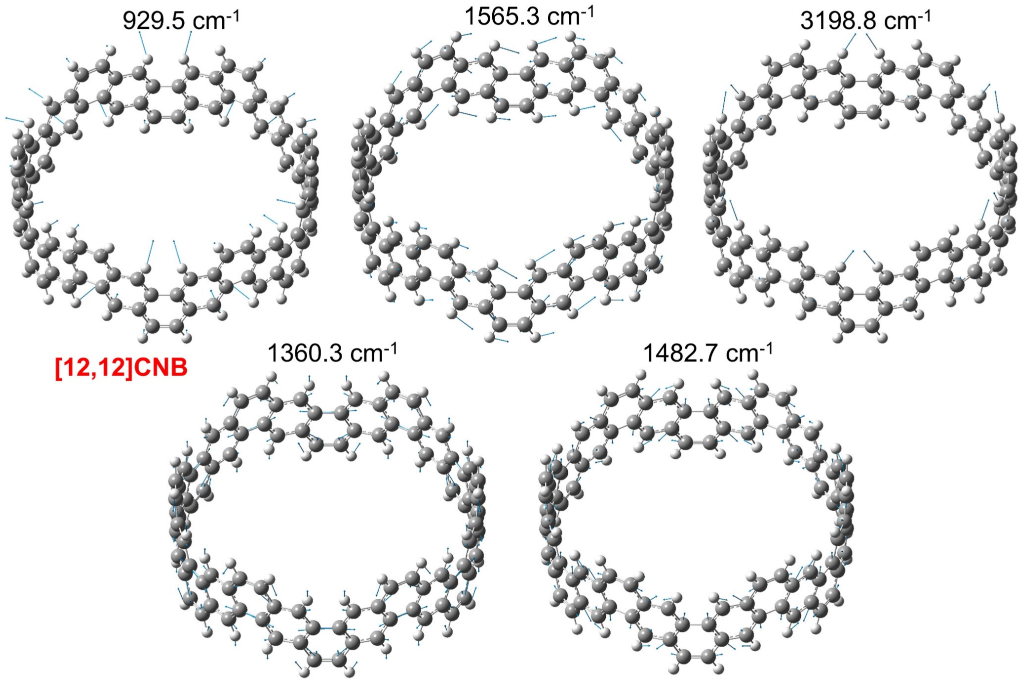

Figure S11. Vibrational modes of [12,12]CNB at 929.5, 1360.3, 1482.7, 1565.3 and 3198.8 cm^{-1}

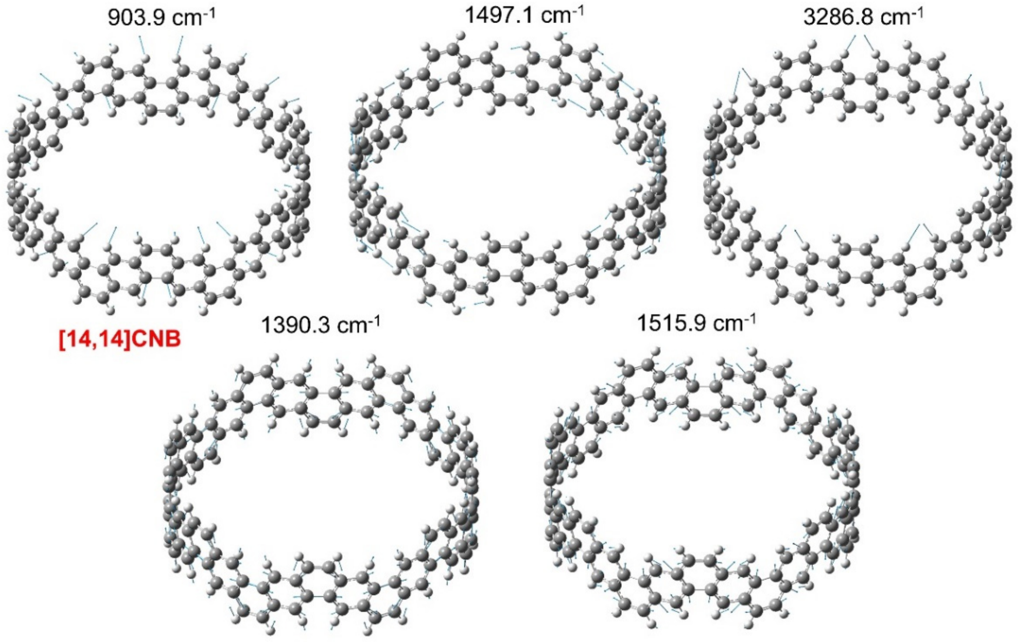

Figure S12. Vibrational modes of [14,14]CNB at 903.9, 1390.3, 1497.1, 1515.9 and 3286.6 cm^{-1}

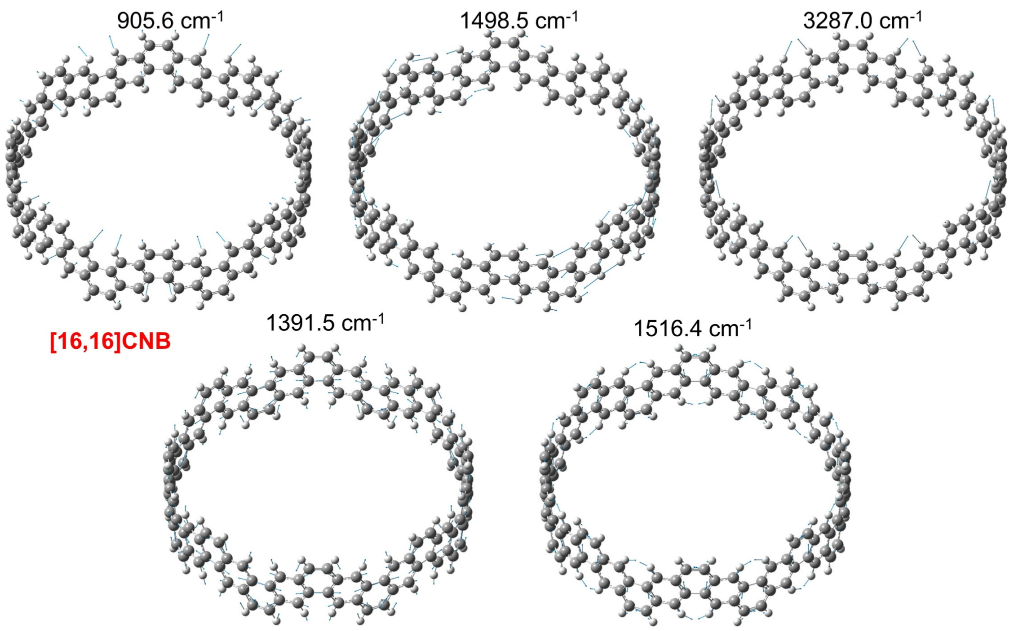

Figure S13. Vibrational modes of [16,16]CNB at 905.6, 1391.5, 1498.5, 1516.4 and 3287.0 cm^{-1}

Coordinates of [2N,2N]CNB

[4,4]CNB

C	-2.75450600	0.65843300	-2.42624800
C	-2.74474300	-0.69736300	-2.42638400
C	2.75452200	-0.65842800	-2.42624100
C	2.74475700	0.69736700	-2.42637600
C	1.44641300	2.41818500	-1.21838900
C	0.72085600	2.72527300	-0.05234100
C	-0.75987200	2.71491200	-0.05217100
C	2.71496800	0.75988300	0.05218900
C	1.35999700	2.42829400	1.19253700
C	0.65843200	2.75441400	2.42624600
C	-1.39437600	2.40847900	1.19277700
C	-0.69737300	2.74464700	2.42638000
C	-2.41825000	1.44641900	1.21838200
C	0.69736600	-2.74465900	2.42637700
C	2.39782500	1.48127800	1.21810800
C	2.41824300	-1.44642100	1.21838600
C	-1.48127100	2.39777200	-1.21811200
C	-2.40849700	-1.39436300	-1.19278200
C	-2.42830400	1.35999100	-1.19253900
C	-2.72532900	0.72086800	0.05235200
C	-1.44641400	-2.41818900	-1.21839500
C	-0.72085900	-2.72527200	-0.05234900
C	-2.71496800	-0.75988100	0.05217800
C	1.48127100	-2.39776400	-1.21811400
C	0.75987100	-2.71491300	-0.05217400
C	2.40849400	1.39436400	-1.19277500
C	2.42830700	-1.35998700	-1.19253400
C	2.72532900	-0.72086900	0.05235900
C	-2.39782600	-1.48127700	1.21810100
C	-1.36000000	-2.42828900	1.19253100
C	-0.65843800	-2.75442100	2.42624000
C	1.39437200	-2.40848500	1.19277500

H	-2.84177700	1.22365300	-3.35794700
H	-2.82385700	-1.26359900	-3.35819300
H	2.84181300	-1.22364800	-3.35793800
H	2.82388900	1.26360600	-3.35818200
H	1.06805600	2.72116300	-2.19685800
H	-1.10766900	2.70660000	-2.19659100
H	-1.06805500	-2.72116200	-2.19686500
H	1.10766900	-2.70658700	-2.19659400
H	1.22363600	2.84159000	3.35796300
H	-1.26359500	2.82366300	3.35820600
H	-2.70664000	-1.10767500	2.19658500
H	-2.72121500	1.06806200	2.19685500
H	-1.22364500	-2.84160500	3.35795500
H	1.26358800	-2.82368700	3.35820200
H	2.70663100	1.10767400	2.19659300
H	2.72120000	-1.06806400	2.19686100

[6,6]CNB

C	2.68540000	3.17180000	1.19330000
C	3.18790000	2.68870000	-0.05710000
C	2.63710000	3.24480000	-1.22080000
C	1.40410000	3.91150000	-1.19330000
C	0.73450000	4.10510000	0.05710000
C	1.49160000	3.90620000	1.22080000
C	-0.73450000	4.10510000	0.05710000
C	-1.40410000	3.91150000	-1.19330000
C	-0.67790000	4.15170000	-2.42200000
C	0.67790000	4.15170000	-2.42200000
C	-1.49160000	3.90620000	1.22080000
C	-2.68540000	3.17180000	1.19330000
C	-3.18790000	2.68870000	-0.05710000
C	-2.63710000	3.24480000	-1.22080000
C	-3.25650000	2.66300000	2.42200000
C	-3.93450000	1.48870000	2.42200000
C	-4.08950000	0.73980000	1.19330000
C	-3.92240000	1.41640000	-0.05710000
C	-4.12860000	-0.66140000	1.22080000
C	-3.92240000	-1.41640000	0.05710000
C	-4.08950000	-0.73980000	-1.19330000
C	-4.12860000	0.66140000	-1.22080000
C	-3.18790000	-2.68870000	0.05710000
C	-2.68540000	-3.17180000	-1.19330000
C	-3.25650000	-2.66300000	-2.42200000
C	-3.93450000	-1.48870000	-2.42200000
C	-2.63710000	-3.24480000	1.22080000
C	-1.40410000	-3.91150000	1.19330000
C	-0.73450000	-4.10510000	-0.05710000
C	-1.49160000	-3.90620000	-1.22080000
C	-0.67790000	-4.15170000	2.42200000
C	0.67790000	-4.15170000	2.42200000
C	1.40410000	-3.91150000	1.19330000
C	0.73450000	-4.10510000	-0.05710000
C	2.63710000	-3.24480000	1.22080000
C	3.18790000	-2.68870000	0.05710000

C	2.68540000	-3.17180000	-1.19330000
C	1.49160000	-3.90620000	-1.22080000
C	3.92240000	-1.41640000	0.05710000
C	4.08950000	-0.73980000	-1.19330000
C	3.93450000	-1.48870000	-2.42200000
C	3.25650000	-2.66300000	-2.42200000
C	4.12860000	-0.66140000	1.22080000
C	4.08950000	0.73980000	1.19330000
C	3.92240000	1.41640000	-0.05710000
C	4.12860000	0.66140000	-1.22080000
C	3.93450000	1.48870000	2.42200000
C	3.25650000	2.66300000	2.42200000
H	4.26100000	1.03420000	3.35420000
H	1.23480000	-4.20720000	3.35420000
H	-1.23480000	-4.20720000	3.35420000
H	-3.02610000	3.17300000	3.35420000
H	-4.26100000	-1.03420000	-3.35420000
H	-3.02610000	-3.17300000	-3.35420000
H	1.23480000	4.20720000	-3.35420000
H	3.02610000	-3.17300000	-3.35420000
H	4.26100000	-1.03420000	-3.35420000
H	-1.23480000	4.20720000	-3.35420000
H	-4.26100000	1.03420000	3.35420000
H	3.02610000	3.17300000	3.35420000
H	-1.06310000	-4.12610000	-2.19450000
H	-3.04180000	-2.98380000	2.19450000
H	1.06310000	-4.12610000	-2.19450000
H	3.04180000	-2.98380000	2.19450000
H	4.10490000	-1.14240000	2.19450000
H	4.10490000	1.14240000	-2.19450000
H	3.04180000	2.98380000	-2.19450000
H	1.06310000	4.12610000	2.19450000
H	-1.06310000	4.12610000	2.19450000
H	-3.04180000	2.98380000	-2.19450000
H	-4.10490000	1.14240000	-2.19450000
H	-4.10490000	-1.14240000	2.19450000

[8,8]CNB

C	-4.36210000	3.42580000	-1.22050000
C	-4.78950000	2.77630000	-0.05570000
C	-4.39550000	3.35070000	1.19590000
C	-3.42580000	4.36210000	1.22050000
C	-2.77630000	4.78950000	0.05570000
C	-3.35070000	4.39550000	-1.19590000
C	-2.74220000	4.84950000	-2.42430000
C	-1.48990000	5.37070000	-2.42440000
C	-0.73870000	5.48170000	-1.19600000
C	-1.42330000	5.35260000	0.05560000
C	0.66210000	5.51160000	-1.22040000
C	1.42330000	5.35260000	-0.05560000
C	0.73870000	5.48170000	1.19600000
C	-0.66210000	5.51160000	1.22040000
C	2.77630000	4.78950000	-0.05570000
C	3.35070000	4.39550000	1.19590000

C	2.74220000	4.84950000	2.42430000
C	1.48990000	5.37070000	2.42440000
C	3.42580000	4.36210000	-1.22050000
C	4.39550000	3.35070000	-1.19590000
C	4.78950000	2.77630000	0.05570000
C	4.36210000	3.42580000	1.22050000
C	4.84950000	2.74220000	-2.42430000
C	5.37070000	1.48990000	-2.42440000
C	5.48170000	0.73870000	-1.19600000
C	5.35260000	1.42330000	0.05560000
C	5.51160000	-0.66210000	-1.22040000
C	5.35260000	-1.42330000	-0.05560000
C	5.48170000	-0.73870000	1.19600000
C	5.51160000	0.66210000	1.22040000
C	4.78950000	-2.77630000	-0.05570000
C	4.39550000	-3.35070000	1.19590000
C	4.84950000	-2.74220000	2.42430000
C	5.37070000	-1.48990000	2.42440000
C	4.36210000	-3.42580000	-1.22050000
C	3.35070000	-4.39550000	-1.19590000
C	2.77630000	-4.78950000	0.05570000
C	3.42580000	-4.36210000	1.22050000
C	2.74220000	-4.84950000	-2.42430000
C	1.48990000	-5.37070000	-2.42440000
C	0.73870000	-5.48170000	-1.19600000
C	1.42330000	-5.35260000	0.05560000
C	-0.66210000	-5.51160000	-1.22040000
C	-1.42330000	-5.35260000	-0.05560000
C	-0.73870000	-5.48170000	1.19600000
C	0.66210000	-5.51160000	1.22040000
C	-2.77630000	-4.78950000	-0.05570000
C	-3.35070000	-4.39550000	1.19590000
C	-2.74220000	-4.84950000	2.42430000
C	-1.48990000	-5.37070000	2.42440000
C	-3.42580000	-4.36210000	-1.22050000
C	-4.39550000	-3.35070000	-1.19590000
C	-4.78950000	-2.77630000	0.05570000
C	-4.36210000	-3.42580000	1.22050000
C	-4.84950000	-2.74220000	-2.42430000
C	-5.37070000	-1.48990000	-2.42440000
C	-5.48170000	-0.73870000	-1.19600000
C	-5.35260000	-1.42330000	0.05560000
C	-5.51160000	0.66210000	-1.22040000
C	-5.35260000	1.42330000	-0.05560000
C	-5.48170000	0.73870000	1.19600000
C	-5.51160000	-0.66210000	1.22040000
C	-5.37070000	1.48990000	2.42440000
C	-4.84950000	2.74220000	2.42430000
H	0.99400000	5.62200000	3.35890000
H	3.27010000	4.67490000	3.35880000
H	5.62200000	-0.99400000	3.35890000
H	4.67490000	-3.27010000	3.35880000
H	-0.99400000	-5.62200000	3.35890000
H	-3.27010000	-4.67490000	3.35880000
H	1.14470000	-5.49600000	2.19320000

H	-1.14470000	-5.49600000	-2.19320000
H	-3.07350000	-4.69200000	-2.19340000
H	-4.69200000	-3.07350000	2.19340000
H	3.07350000	-4.69200000	2.19340000
H	4.69200000	-3.07350000	-2.19340000
H	-5.62200000	0.99400000	3.35890000
H	-4.67490000	3.27010000	3.35880000
H	5.62200000	0.99400000	-3.35890000
H	4.67490000	3.27010000	-3.35880000
H	-0.99400000	5.62200000	-3.35890000
H	-3.27010000	4.67490000	-3.35880000
H	-5.62200000	-0.99400000	-3.35890000
H	-4.67490000	-3.27010000	-3.35880000
H	0.99400000	-5.62200000	-3.35890000
H	3.27010000	-4.67490000	-3.35880000
H	5.49600000	1.14470000	2.19320000
H	5.49600000	-1.14470000	-2.19320000
H	4.69200000	3.07350000	2.19340000
H	3.07350000	4.69200000	-2.19340000
H	-1.14470000	5.49600000	2.19320000
H	1.14470000	5.49600000	-2.19320000
H	-3.07350000	4.69200000	2.19340000
H	-4.69200000	3.07350000	-2.19340000
H	-5.49600000	-1.14470000	2.19320000
H	-5.49600000	1.14470000	-2.19320000

[10,10]CNB

C	0.09830000	6.93730000	2.42590000
C	1.44670000	6.78790000	2.42590000
C	2.16670000	6.55720000	1.19730000
C	1.49640000	6.74640000	-0.05450000
C	0.04060000	6.90730000	-0.05460000
C	-0.65470000	6.86970000	1.19730000
C	-2.03160000	6.61060000	1.22010000
C	-2.75460000	6.33210000	0.05470000
C	-2.10090000	6.57170000	-1.19720000
C	-0.73370000	6.87740000	-1.22010000
C	-4.02860000	5.60930000	0.05470000
C	-4.56980000	5.17110000	-1.19720000
C	-3.99980000	5.66790000	-2.42570000
C	-2.81950000	6.33700000	-2.42580000
C	-4.63820000	5.13110000	1.22020000
C	-5.56560000	4.08100000	1.19740000
C	-5.95360000	3.50290000	-0.05450000
C	-5.53260000	4.15340000	-1.22000000
C	-6.00940000	3.46880000	2.42600000
C	-6.57100000	2.23370000	2.42590000
C	-6.73990000	1.49690000	1.19730000
C	-6.55930000	2.16950000	-0.05460000
C	-6.92080000	0.10760000	1.22000000
C	-6.87900000	-0.66620000	0.05460000
C	-6.90410000	0.02950000	-1.19730000
C	-6.77110000	1.42420000	-1.22010000
C	-6.58580000	-2.10140000	0.05470000

C	-6.33650000	-2.75170000	-1.19710000
C	-6.63190000	-2.05590000	-2.42580000
C	-6.90280000	-0.72650000	-2.42590000
C	-6.32020000	-2.82910000	1.22030000
C	-5.60820000	-4.03580000	1.19770000
C	-5.17800000	-4.58340000	-0.05410000
C	-5.66660000	-3.98210000	-1.21970000
C	-5.16290000	-4.64690000	2.42630000
C	-4.16110000	-5.56180000	2.42620000
C	-3.51180000	-5.94960000	1.19760000
C	-4.09640000	-5.57090000	-0.05420000
C	-2.24540000	-6.54880000	1.22020000
C	-1.49640000	-6.74640000	0.05450000
C	-2.16670000	-6.55720000	-1.19730000
C	-3.45300000	-6.00220000	-1.21980000
C	-0.04060000	-6.90730000	0.05460000
C	0.65470000	-6.86970000	-1.19730000
C	-0.09830000	-6.93730000	-2.42590000
C	-1.44670000	-6.78790000	-2.42590000
C	0.73370000	-6.87740000	1.22010000
C	2.10090000	-6.57170000	1.19720000
C	2.75460000	-6.33210000	-0.05470000
C	2.03160000	-6.61060000	-1.22010000
C	2.81950000	-6.33700000	2.42580000
C	3.99980000	-5.66790000	2.42570000
C	4.56980000	-5.17110000	1.19720000
C	4.02860000	-5.60930000	-0.05470000
C	5.53260000	-4.15340000	1.22000000
C	5.95360000	-3.50290000	0.05450000
C	5.56560000	-4.08100000	-1.19740000
C	4.63820000	-5.13110000	-1.22020000
C	6.55930000	-2.16950000	0.05460000
C	6.73990000	-1.49690000	-1.19730000
C	6.57100000	-2.23370000	-2.42590000
C	6.00940000	-3.46880000	-2.42600000
C	6.77110000	-1.42420000	1.22010000
C	6.90410000	-0.02950000	1.19730000
C	6.87900000	0.66620000	-0.05460000
C	6.92080000	-0.10760000	-1.22000000
C	6.90280000	0.72650000	2.42590000
C	6.63190000	2.05590000	2.42580000
C	6.33650000	2.75170000	1.19710000
C	6.58580000	2.10140000	-0.05470000
C	5.66660000	3.98210000	1.21970000
C	5.17800000	4.58340000	0.05410000
C	5.60820000	4.03580000	-1.19770000
C	6.32020000	2.82910000	-1.22030000
C	4.09640000	5.57090000	0.05420000
C	3.51180000	5.94960000	-1.19760000
C	4.16110000	5.56180000	-2.42620000
H	6.55330000	2.60460000	3.36120000
C	3.45300000	6.00220000	1.21980000
C	2.24540000	6.54880000	-1.22020000
H	7.04540000	0.19100000	3.36150000
H	4.49750000	-5.42380000	3.36120000

H	2.35450000	-6.63870000	3.36130000
H	-3.77500000	-5.95970000	3.36160000
H	-5.59400000	-4.29850000	3.36170000
H	-6.83050000	1.74370000	3.36130000
H	-5.81090000	3.98630000	3.36140000
H	-0.44810000	7.03070000	3.36140000
H	2.00020000	6.75960000	3.36140000
C	5.16290000	4.64690000	-2.42630000
H	5.59400000	4.29850000	-3.36170000
H	3.77500000	5.95970000	-3.36160000
H	-2.35450000	6.63870000	-3.36130000
H	-4.49750000	5.42380000	-3.36120000
H	-7.04540000	-0.19100000	-3.36150000
H	-6.55330000	-2.60460000	-3.36120000
H	-2.00020000	-6.75960000	-3.36140000
H	0.44810000	-7.03070000	-3.36140000
H	-6.70460000	1.90290000	-2.19250000
H	-6.96450000	-0.37380000	2.19240000
H	-5.85020000	3.78900000	-2.19240000
H	-4.30380000	5.48010000	2.19270000
H	-5.41800000	-4.39700000	-2.19200000
H	-6.54870000	-2.40300000	2.19270000
H	-1.79990000	-6.73770000	2.19240000
H	-3.88890000	-5.79270000	-2.19220000
H	0.25810000	-6.96330000	2.19260000
H	2.50230000	-6.50140000	-2.19260000
H	4.30380000	-5.48010000	-2.19270000
H	5.85020000	-3.78900000	2.19240000
H	6.70460000	-1.90290000	2.19250000
H	6.96450000	0.37380000	-2.19240000
H	6.54870000	2.40300000	-2.19270000
H	5.41800000	4.39700000	2.19200000
H	3.88890000	5.79270000	2.19220000
H	1.79990000	6.73770000	-2.19240000
H	5.81090000	-3.98630000	-3.36140000
H	6.83050000	-1.74370000	-3.36130000
H	-0.25810000	6.96330000	-2.19260000
H	-2.50230000	6.50140000	2.19260000

[12,12]CNB

C	-7.50980000	-3.55670000	2.43000000
C	-7.77150000	-2.84840000	1.20180000
C	-7.51320000	-3.49380000	-0.05080000
C	-6.78420000	-4.76380000	-0.05220000
C	-6.35660000	-5.31520000	1.19890000
C	-6.83550000	-4.73420000	2.42860000
C	-5.38080000	-6.32040000	1.21900000
C	-4.76380000	-6.78420000	0.05220000
C	-5.31520000	-6.35660000	-1.19890000
C	-6.32040000	-5.38080000	-1.21900000
C	-3.49380000	-7.51320000	0.05080000
C	-2.84840000	-7.77150000	-1.20180000
C	-3.55670000	-7.50980000	-2.43000000
C	-4.73420000	-6.83550000	-2.42860000

C	-2.78080000	-7.81500000	1.21600000
C	-1.42200000	-8.15560000	1.19340000
C	-0.73210000	-8.24650000	-0.05880000
C	-1.49960000	-8.14990000	-1.22450000
C	-0.67840000	-8.28390000	2.42190000
C	0.67840000	-8.28390000	2.42190000
C	1.42200000	-8.15560000	1.19340000
C	0.73210000	-8.24650000	-0.05880000
C	2.78080000	-7.81500000	1.21600000
C	3.49380000	-7.51320000	0.05080000
C	2.84840000	-7.77150000	-1.20180000
C	1.49960000	-8.14990000	-1.22450000
C	4.76380000	-6.78420000	0.05220000
C	5.31520000	-6.35660000	-1.19890000
C	4.73420000	-6.83550000	-2.42860000
C	3.55670000	-7.50980000	-2.43000000
C	5.38080000	-6.32040000	1.21900000
C	6.35660000	-5.31520000	1.19890000
C	6.78420000	-4.76380000	-0.05220000
C	6.32040000	-5.38080000	-1.21900000
C	6.83550000	-4.73420000	2.42860000
C	7.50980000	-3.55670000	2.43000000
C	7.77150000	-2.84840000	1.20180000
C	7.51320000	-3.49380000	-0.05080000
C	8.14990000	-1.49960000	1.22450000
C	8.24650000	-0.73210000	0.05880000
C	8.15560000	-1.42200000	-1.19340000
C	7.81500000	-2.78080000	-1.21600000
C	8.24650000	0.73210000	0.05880000
C	8.15560000	1.42200000	-1.19340000
C	8.28390000	0.67840000	-2.42190000
C	8.28390000	-0.67840000	-2.42190000
C	8.14990000	1.49960000	1.22450000
C	7.77150000	2.84840000	1.20180000
C	7.51320000	3.49380000	-0.05080000
C	7.81500000	2.78080000	-1.21600000
C	7.50980000	3.55670000	2.43000000
C	6.83550000	4.73420000	2.42860000
C	6.35660000	5.31520000	1.19890000
C	6.78420000	4.76380000	-0.05220000
C	5.38080000	6.32040000	1.21900000
C	4.76380000	6.78420000	0.05220000
C	5.31520000	6.35660000	-1.19890000
C	6.32040000	5.38080000	-1.21900000
C	3.49380000	7.51320000	0.05080000
C	2.84840000	7.77150000	-1.20180000
C	3.55670000	7.50980000	-2.43000000
C	4.73420000	6.83550000	-2.42860000
C	2.78080000	7.81500000	1.21600000
C	1.42200000	8.15560000	1.19340000
C	0.73210000	8.24650000	-0.05880000
C	1.49960000	8.14990000	-1.22450000
C	0.67840000	8.28390000	2.42190000
C	-0.67840000	8.28390000	2.42190000
C	-1.42200000	8.15560000	1.19340000

C	-0.73210000	8.24650000	-0.05880000
C	-2.78080000	7.81500000	1.21600000
C	-3.49380000	7.51320000	0.05080000
C	-2.84840000	7.77150000	-1.20180000
C	-1.49960000	8.14990000	-1.22450000
C	-4.76380000	6.78420000	0.05220000
C	-5.31520000	6.35660000	-1.19890000
C	-4.73420000	6.83550000	-2.42860000
C	-3.55670000	7.50980000	-2.43000000
C	-5.38080000	6.32040000	1.21900000
C	-6.35660000	5.31520000	1.19890000
C	-6.78420000	4.76380000	-0.05220000
C	-6.32040000	5.38080000	-1.21900000
C	-6.83550000	4.73420000	2.42860000
C	-7.50980000	3.55670000	2.43000000
C	-7.77150000	2.84840000	1.20180000
C	-7.51320000	3.49380000	-0.05080000
C	-8.14990000	1.49960000	1.22450000
C	-8.24650000	0.73210000	0.05880000
C	-8.15560000	1.42200000	-1.19340000
C	-7.81500000	2.78080000	-1.21600000
C	-8.24650000	-0.73210000	0.05880000
C	-8.15560000	-1.42200000	-1.19340000
C	-8.28390000	-0.67840000	-2.42190000
C	-8.28390000	0.67840000	-2.42190000
C	-8.14990000	-1.49960000	1.22450000
C	-7.81500000	-2.78080000	-1.21600000
H	6.58340000	5.22790000	3.36400000
H	7.80730000	3.09130000	3.36650000
H	7.80730000	-3.09130000	3.36650000
H	6.58340000	-5.22790000	3.36400000
H	1.23120000	-8.31350000	3.35780000
H	-1.23120000	-8.31350000	3.35780000
H	-6.58340000	-5.22790000	3.36400000
H	-7.80730000	-3.09130000	3.36650000
H	-7.80730000	3.09130000	3.36650000
H	-6.58340000	5.22790000	3.36400000
H	-1.23120000	8.31350000	3.35780000
H	1.23120000	8.31350000	3.35780000
H	-5.22790000	-6.58340000	-3.36400000
H	-3.09130000	-7.80730000	-3.36650000
H	3.09130000	-7.80730000	-3.36650000
H	5.22790000	-6.58340000	-3.36400000
H	8.31350000	-1.23120000	-3.35780000
H	8.31350000	1.23120000	-3.35780000
H	5.22790000	6.58340000	-3.36400000
H	3.09130000	7.80730000	-3.36650000
H	-3.09130000	7.80730000	-3.36650000
H	-5.22790000	6.58340000	-3.36400000
H	-8.31350000	1.23120000	-3.35780000
H	-8.31350000	-1.23120000	-3.35780000
H	-7.68340000	3.24540000	-2.18860000
H	-8.26240000	1.02970000	2.19700000
H	-6.65690000	5.03170000	-2.19060000
H	-5.03170000	6.65690000	2.19060000

H	-8.26240000	-1.02970000	2.19700000
H	-7.68340000	-3.24540000	-2.18860000
H	-5.03170000	-6.65690000	2.19060000
H	-6.65690000	-5.03170000	-2.19060000
H	-3.24540000	-7.68340000	2.18860000
H	-1.02970000	-8.26240000	-2.19700000
H	1.02970000	-8.26240000	-2.19700000
H	3.24540000	-7.68340000	2.18860000
H	5.03170000	-6.65690000	2.19060000
H	6.65690000	-5.03170000	-2.19060000
H	7.68340000	-3.24540000	-2.18860000
H	8.26240000	-1.02970000	2.19700000
H	8.26240000	1.02970000	2.19700000
H	7.68340000	3.24540000	-2.18860000
H	6.65690000	5.03170000	-2.19060000
H	5.03170000	6.65690000	2.19060000
H	3.24540000	7.68340000	2.18860000
H	1.02970000	8.26240000	-2.19700000
H	-3.24540000	7.68340000	2.18860000
H	-1.02970000	8.26240000	-2.19700000

[14,14]CNB

C	-8.98714800	-3.58704400	2.43251600
C	-9.40098000	-2.29244100	2.43249300
C	-9.52639300	-1.55032000	1.20241900
C	-9.39356900	-2.23560800	-0.04977500
C	-8.94816500	-3.62913300	-0.04975900
C	-8.65885000	-4.26436100	1.20246400
C	-7.96015500	-5.48010400	1.21986500
C	-7.49360400	-6.09009700	0.04962800
C	-7.91059900	-5.53036000	-1.20259200
C	-8.63876900	-4.33203400	-1.22000100
C	-6.48758300	-7.15226100	0.04969300
C	-5.95122300	-7.59909100	-1.20247000
C	-6.54093900	-7.13144800	-2.43256200
C	-7.47554500	-6.14464300	-2.43262000
C	-5.90376500	-7.65104000	1.21999000
C	-4.72768400	-8.41461700	1.20269600
C	-4.10914500	-8.73823200	-0.04948400
C	-4.79419700	-8.39125100	-1.21976500
C	-4.06906900	-8.77885200	2.43278700
C	-2.79877300	-9.26218200	2.43279200
C	-2.06462800	-9.42794300	1.20270800
C	-2.74182500	-9.25852800	-0.04947700
C	-0.67845100	-9.63943600	1.21999900
C	0.08924600	-9.65511100	0.04969700
C	-0.60847600	-9.63231300	-1.20246300
C	-1.99942000	-9.45480200	-1.21975200
C	1.54693400	-9.53083500	0.04963000
C	2.23058600	-9.39016400	-1.20259200
C	1.49716200	-9.55959600	-2.43261300
C	0.14293800	-9.67512800	-2.43255200
C	2.30103600	-9.38561500	1.21986100
C	3.63138900	-8.94248500	1.20246200

C	4.26993700	-8.66057800	-0.04976200
C	3.57136700	-8.97966700	-1.22000000
C	4.32703600	-8.65506900	2.43251500
C	5.49711700	-7.96357400	2.43249000
C	6.08433500	-7.49277200	1.20241700
C	5.52939700	-7.91622300	-0.04978200
C	7.11417000	-6.54111500	1.21979000
C	7.60499800	-5.95047100	0.04955600
C	7.15195200	-6.48147900	-1.20264900
C	6.14574300	-7.45808100	-1.22002700
C	8.41672100	-4.73334200	0.04960400
C	8.73287000	-4.11094400	-1.20256700
C	8.40804600	-4.78986900	-2.43265900
C	7.65394500	-5.92062200	-2.43269700
C	8.77327900	-4.05325200	1.21989400
C	9.25593100	-2.73671900	1.20260200
C	9.43353800	-2.06159600	-0.04956900
C	9.24763900	-2.80663600	-1.21985900
C	9.46469200	-2.01361500	2.43268700
C	9.65318000	-0.66760000	2.43272600
C	9.65117800	0.08508700	1.20268000
C	9.63649900	-0.61276800	-0.04952400
C	9.54910400	1.48358600	1.22003500
C	9.39357000	2.23560900	0.04977800
C	9.52639300	1.55031900	-1.20241700
C	9.66264000	0.15473700	-1.21976900
C	8.94816600	3.62913500	0.04976100
C	8.65885000	4.26436300	-1.20246400
C	8.98715100	3.58704400	-2.43251600
C	9.40098300	2.29244200	-2.43249100
C	8.63877100	4.33203600	1.22000100
C	7.91059900	5.53036200	1.20259200
C	7.49360400	6.09009800	-0.04962800
C	7.96015500	5.48010400	-1.21986600
C	7.47554600	6.14464500	2.43262000
C	6.54094000	7.13145200	2.43256000
C	5.95122700	7.59909400	1.20246800
C	6.48758400	7.15226000	-0.04969300
C	4.79420000	8.39125600	1.21976400
C	4.10914800	8.73823500	0.04948400
C	4.72768500	8.41461600	-1.20269600
C	5.90376400	7.65103900	-1.21999200
C	2.74182500	9.25853000	0.04947600
C	2.06462700	9.42794000	-1.20271000
C	2.79877400	9.26218000	-2.43279600
C	4.06907000	8.77885100	-2.43278900
C	1.99942200	9.45480500	1.21975000
C	0.60847600	9.63231300	1.20246300
C	-0.08924600	9.65510700	-0.04969700
C	0.67845300	9.63943000	-1.22000100
C	-0.14293600	9.67512700	2.43255000
C	-1.49716200	9.55959400	2.43261000
C	-2.23058400	9.39016300	1.20259100
C	-1.54693100	9.53083200	-0.04963100
C	-3.57136800	8.97966700	1.21999800

C	-4.26993700	8.66057800	0.04976300
C	-3.63138700	8.94248300	-1.20246200
C	-2.30103500	9.38561100	-1.21986300
C	-5.52939900	7.91622200	0.04978200
C	-6.08433500	7.49276800	-1.20241800
C	-5.49711600	7.96357200	-2.43249200
C	-4.32703600	8.65506700	-2.43251600
C	-6.14574400	7.45808400	1.22002600
C	-7.15195400	6.48148000	1.20265000
C	-7.60499800	5.95047000	-0.04955400
C	-7.11416800	6.54111200	-1.21979000
C	-7.65394700	5.92062400	2.43269700
C	-8.40804700	4.78986900	2.43265900
C	-8.73287200	4.11094500	1.20256900
C	-8.41672100	4.73334300	-0.04960300
C	-9.24764200	2.80663700	1.21986100
C	-9.43354100	2.06159700	0.04957100
C	-9.25593400	2.73671900	-1.20259900
C	-8.77328200	4.05325200	-1.21989200
C	-9.63650100	0.61276800	0.04952700
C	-9.65118100	-0.08508700	-1.20267900
C	-9.65318500	0.66760200	-2.43272400
C	-9.46469700	2.01361600	-2.43268600
C	-9.66264100	-0.15473600	1.21977100
C	-9.54910500	-1.48358400	-1.22003400
H	5.98632300	-7.69994900	3.37358300
H	3.86016700	-8.95642400	3.37362600
H	-2.28751900	-9.47990500	3.37390200
H	-4.59575500	-8.60166500	3.37389300
H	-8.83894100	-4.12259700	3.37362600
H	-9.59095600	-1.77020900	3.37358600
H	-8.73435400	4.34003600	3.37374600
H	-7.36413100	6.39473300	3.37381500
H	-2.05224100	9.53430200	3.37373700
H	0.40848700	9.74426900	3.37362500
H	6.17509300	7.54979400	3.37363200
H	7.87332200	5.75665700	3.37374200
H	9.75188600	-0.12077100	3.37384600
H	9.40932100	-2.56656600	3.37377900
H	7.46459600	-6.19745300	2.19401600
H	8.59062300	-4.50889000	2.19408500
H	-0.19115200	-9.69871800	2.19418900
H	1.83092200	-9.52667100	2.19409000
H	-7.70296200	-5.89812900	2.19410200
H	-6.30723200	-7.37141000	2.19418200
H	-9.41408600	2.34484400	2.19405700
H	-9.69581700	0.33498300	2.19398600
H	-4.03616100	8.82213200	2.19424500
H	-5.78351700	7.78925400	2.19425300
H	4.38111500	8.65641900	2.19396600
H	2.48429100	9.37821700	2.19394600
H	9.49871100	1.97182000	2.19425100
H	8.88106400	3.90524100	2.19424600
H	9.41408300	-2.34484200	-2.19405500
H	8.73435600	-4.34003300	-3.37374500

H	5.78351600	-7.78925100	-2.19425400
H	7.36412800	-6.39473500	-3.37381300
H	4.03616300	-8.82213000	-2.19424400
H	2.05224500	-9.53430600	-3.37373700
H	-2.48428800	-9.37821400	-2.19394800
H	-0.40849000	-9.74427300	-3.37362400
H	-4.38110900	-8.65641400	-2.19396600
H	-6.17509200	-7.54979000	-3.37363400
H	-8.88106300	-3.90524000	-2.19424600
H	-7.87332300	-5.75665500	-3.37374200
H	-9.49871200	-1.97182100	-2.19424800
H	-9.75189300	0.12077200	-3.37384400
H	-8.59062700	4.50888800	-2.19408400
H	-9.40932900	2.56656800	-3.37377600
H	-7.46459600	6.19744600	-2.19401500
H	-5.98632300	7.69994800	-3.37358400
H	-1.83092200	9.52666700	-2.19409200
H	-3.86016600	8.95642300	-3.37362700
H	0.19114900	9.69871100	-2.19418900
H	2.28751700	9.47990500	-3.37390400
H	6.30723000	7.37140700	-2.19418400
H	4.59576000	8.60166400	-3.37389200
H	7.70296200	5.89813100	-2.19410100
H	8.83894600	4.12259800	-3.37362600
H	9.69581700	-0.33498200	-2.19398400
H	9.59096200	1.77020900	-3.37358400

[16,16]CNB

C	0.42874100	11.02447000	0.04935600
C	1.19447700	10.97502200	1.21972900
C	2.57404800	10.72407600	1.20278300
C	3.24813900	10.54288400	-0.04945000
C	2.50935400	10.75021200	-1.21984200
C	1.12471800	10.97146300	-1.20288800
C	-1.03358300	10.98443200	0.04940200
C	-1.72571500	10.89350500	-1.20280500
C	-0.98243400	11.00718300	-2.43295800
C	0.37627200	11.04434700	-2.43299500
C	-1.79538000	10.89300000	1.21982800
C	-3.15912600	10.56683000	1.20295300
C	-3.82235100	10.34913700	-0.04925600
C	-3.09617000	10.59677400	-1.21967600
C	-3.87835700	10.34738800	2.43307300
C	-5.11921700	9.79264800	2.43306200
C	-5.76233200	9.40299900	1.20293200
C	-5.15779900	9.75202300	-0.04927300
C	-6.91483100	8.60429300	1.21981100
C	-7.49100700	8.09764500	0.04938600
C	-6.96161000	8.55264700	-1.20282700
C	-5.82651400	9.37589000	-1.21971100
C	-8.49648700	7.03509900	0.04934800
C	-8.92158800	6.48147600	-1.20288500
C	-8.47643700	7.08754600	-2.43299500
C	-7.54216800	8.07475800	-2.43296700

C	-8.97060100	6.43179400	1.21973400
C	-9.70475900	5.23713900	1.20278800
C	10.01998500	4.61435700	-0.04944500
C	-9.68114200	5.30280400	-1.21983400
C	10.05865700	4.57366200	2.43290200
C	10.54456900	3.30427100	2.43290300
C	10.72408200	2.57404700	1.20278700
C	10.54289100	3.24814000	-0.04944600
C	10.97502800	1.19447700	1.21973200
C	11.02447500	0.42874200	0.04935900
C	10.97146800	1.12471900	-1.20288500
C	10.75021800	2.50935600	-1.21983800
C	10.98443600	-1.03358100	0.04940300
C	10.89350900	-1.72571200	-1.20280200
C	11.00718500	-0.98243300	-2.43295400
C	11.04434900	0.37627300	-2.43299200
C	10.89300200	-1.79538000	1.21983000
C	10.56683200	-3.15912400	1.20295400
C	10.34914000	-3.82235100	-0.04925700
C	10.59677700	-3.09617000	-1.21967500
C	10.34738900	-3.87835700	2.43307300
C	-9.79264800	-5.11921700	2.43306200
C	-9.40299900	-5.76233200	1.20293100
C	-9.75202500	-5.15780000	-0.04927300
C	-8.60429200	-6.91483000	1.21980700
C	-8.09764600	-7.49100500	0.04938000
C	-8.55264900	-6.96160700	-1.20283200
C	-9.37589400	-5.82651100	-1.21971300
C	-7.03510000	-8.49648400	0.04934000
C	-6.48147700	-8.92158300	-1.20289400
C	-7.08754900	-8.47643100	-2.43300300
C	-8.07476100	-7.54216200	-2.43297300
C	-6.43179400	-8.97060100	1.21972400
C	-5.23713900	-9.70475800	1.20277700
C	-4.61435700	-10.01998200	-0.04945700
C	-5.30280600	-9.68113700	-1.21984500
C	-4.57366000	-10.05865700	2.43289000
C	-3.30427000	-10.54456900	2.43288900
C	-2.57404700	-10.72408000	1.20277200
C	-3.24814000	-10.54288700	-0.04946000
C	-1.19447700	-10.97502600	1.21971500
C	-0.42874200	-11.02447100	0.04934100
C	-1.12472100	-10.97146200	-1.20290100
C	-2.50935700	-10.75021300	-1.21985300
C	1.03358200	-10.98443200	0.04938600
C	1.72571200	-10.89350200	-1.20282200
C	0.98243000	-11.00717800	-2.43297400
C	-0.37627600	-11.04434300	-2.43301000
C	1.79538100	-10.89300200	1.21981000
C	3.15912600	-10.56683100	1.20293500
C	3.82235000	-10.34913500	-0.04927400
C	3.09616700	-10.59676900	-1.21969400
C	3.87835800	-10.34739300	2.43305500
C	5.11921800	-9.79265200	2.43304400
C	5.76233100	-9.40300000	1.20291400

C	5.15779700	-9.75202100	-0.04929100
C	6.91482900	-8.60429200	1.21979400
C	7.49100500	-8.09764100	0.04937000
C	6.96160700	-8.55264100	-1.20284400
C	5.82651000	-9.37588500	-1.21972900
C	8.49648400	-7.03509600	0.04933300
C	8.92158600	-6.48147200	-1.20289900
C	8.47643600	-7.08754000	-2.43301000
C	7.54216500	-8.07475100	-2.43298300
C	8.97059600	-6.43178900	1.21972000
C	9.70475500	-5.23713600	1.20277500
C	10.01998500	-4.61435600	-0.04945600
C	9.68114300	-5.30280200	-1.21984600
C	10.05864600	-4.57365900	2.43289400
C	10.54455800	-3.30427100	2.43289700
C	10.72407800	-2.57404700	1.20277600
C	10.54289300	-3.24813800	-0.04945600
C	10.97502400	-1.19447800	1.21972500
C	11.02447900	-0.42874100	0.04935200
C	10.97147800	-1.12471800	-1.20289200
C	10.75022700	-2.50935500	-1.21984700
C	10.98444000	1.03358200	0.04939800
C	10.89351600	1.72571400	-1.20281000
C	11.00719900	0.98243300	-2.43296200
C	11.04436600	-0.37627200	-2.43299900
C	10.89300700	1.79538000	1.21982300
C	10.56683600	3.15912500	1.20294800
C	10.34914400	3.82235000	-0.04926200
C	10.59678400	3.09616900	-1.21968200
C	10.34739400	3.87835700	2.43306700
C	9.79265400	5.11921700	2.43305600
C	9.40300400	5.76233000	1.20292600
C	9.75202700	5.15779700	-0.04927900
C	8.60429600	6.91482800	1.21980400
C	8.09764600	7.49100200	0.04937900
C	8.55264600	6.96160400	-1.20283400
C	9.37589100	5.82650900	-1.21971800
C	7.03509900	8.49648100	0.04934100
C	6.48147300	8.92158000	-1.20289200
C	7.08754200	8.47642800	-2.43300200
C	8.07475400	7.54215900	-2.43297400
C	6.43179400	8.97059600	1.21972700
C	5.23713900	9.70475300	1.20278200
C	4.61435500	10.01997800	-0.04945000
C	5.30280200	9.68113400	-1.21984000
C	4.57366200	10.05865000	2.43289700
C	3.30427200	10.54456100	2.43289800
H	10.66007600	-2.99194800	-2.19394700
H	11.07855400	-0.93052300	-3.37430800
H	11.01100200	1.53777300	-3.37424000
H	9.65356200	5.42152700	-2.19378100
H	10.48020800	3.57318300	-2.19373300
H	8.49101100	7.17454100	-3.37425500
H	6.69763600	8.87183600	-3.37430300
H	2.99194600	10.66005600	-2.19394200

H	4.88334500	9.93627900	-2.19393100
H	0.93052300	11.07853000	-3.37430400
H	-1.53777400	11.01098300	-3.37423600
H	-5.42153300	9.65356300	-2.19377400
H	-3.57318300	10.48019600	-2.19372800
H	-7.17455000	8.49101500	-3.37424700
H	-8.87184700	6.69764200	-3.37429600
H	10.66006100	2.99194900	-2.19393800
H	-9.93628800	4.88334900	-2.19392500
H	11.07853100	0.93052300	-3.37430200
H	11.01098400	-1.53777000	-3.37423400
H	-9.65357000	-5.42152700	-2.19377300
H	10.48019800	-3.57317500	-2.19373100
H	-8.49102000	-7.17454400	-3.37425200
H	-6.69764500	-8.87183900	-3.37430500
H	-2.99195100	-10.66005500	-2.19395200
H	-4.88335100	-9.93628100	-2.19393700
H	-0.93052800	-11.07852500	-3.37431800
H	1.53776800	-11.01097600	-3.37425300
H	5.42153000	-9.65355600	-2.19379200
H	3.57317900	-10.48018800	-2.19374600
H	7.17454600	-8.49100600	-3.37426400
H	8.87184600	-6.69763500	-3.37431000
H	9.93629000	-4.88334500	-2.19393600
H	11.05033900	-0.70933300	2.19381700
H	10.99457700	1.31501400	2.19389500
H	8.31416600	7.31093300	2.19387900
H	6.84328300	8.70277800	2.19381600
H	0.70933500	11.05034200	2.19382100
H	-1.31501400	10.99457000	2.19389900
H	-7.31093600	8.31416300	2.19388600
H	-8.70278300	6.84328200	2.19382300
H	11.05034900	0.70933400	2.19382400
H	10.99457200	-1.31501400	2.19390200
H	-8.31416000	-7.31093600	2.19388100
H	-6.84328100	-8.70278400	2.19381500
H	-0.70933300	-11.05034800	2.19380700
H	1.31501500	-10.99457500	2.19388200
H	7.31093600	-8.31416600	2.19387000
H	8.70277300	-6.84327700	2.19380800
H	10.76092800	-2.79282000	3.37418600
H	10.59119800	3.37943300	3.37437300
H	9.58343900	5.63362200	3.37435200
H	5.09887300	9.87824500	3.37418900
H	2.79283300	10.76093000	3.37419500
H	-3.37943200	10.59119100	3.37437900
H	-5.63362100	9.58343200	3.37435900
H	-9.87825200	5.09887200	3.37419500
H	10.76093800	2.79283200	3.37419900
H	10.59119100	-3.37943300	3.37437900
H	-9.58343200	-5.63362000	3.37435800
H	-5.09887000	-9.87825500	3.37418300
H	-2.79283000	-10.76093900	3.37418400
H	3.37943500	-10.59119800	3.37436000
H	5.63362300	-9.58343900	3.37434000

H	9.87823700	-5.09887600	3.37418200
